# Concordance between RNA-sequencing data and DNA microarray data in transcriptome analysis of proliferative and quiescent fibroblasts

**DOI:** 10.1098/rsos.150402

**Published:** 2015-09-30

**Authors:** Brett Trost, Catherine A. Moir, Zoe E. Gillespie, Anthony Kusalik, Jennifer A. Mitchell, Christopher H. Eskiw

**Affiliations:** 1 Department of Computer Science, University of Saskatchewan, Saskatoon Canada S7N 5C9; 2 Department of Life Sciences, Brunel University, Uxbridge UB8 3PH, UK; 3 Nuclear Dynamics Programme, Babraham Institute, Cambridge CB22 3AT, UK; 4 Department of Food and Bioproduct Sciences, University of Saskatchewan, Saskatoon Canada S7N 5A8; 5 Department of Cell and Systems Biology, University of Toronto, Toronto, Canada M5S 3G5; 6 Centre for the Analysis of Genome Evolution and Function, University of Toronto, Toronto Canada M5S 3G5

**Keywords:** RNA-seq, microarrays, transcriptome analysis, fibroblasts, gene expression

## Abstract

DNA microarrays and RNA sequencing (RNA-seq) are major technologies for performing high-throughput analysis of transcript abundance. Recently, concerns have been raised regarding the concordance of data derived from the two techniques. Using cDNA libraries derived from normal human foreskin fibroblasts, we measured changes in transcript abundance as cells transitioned from proliferative growth to quiescence using both DNA microarrays and RNA-seq. The internal reproducibility of the RNA-seq data was greater than that of the microarray data. Correlations between the RNA-seq data and the individual microarrays were low, but correlations between the RNA-seq values and the geometric mean of the microarray values were moderate. The two technologies had good agreement when considering probes with the largest (both positive and negative) fold change (FC) values. An independent technique, quantitative reverse-transcription PCR (qRT-PCR), was used to measure the FC of 76 genes between proliferative and quiescent samples, and a higher correlation was observed between the qRT-PCR data and the RNA-seq data than between the qRT-PCR data and the microarray data.

## 1. Introduction

Since being introduced in the mid-1990s [1], the DNA microarray has become a highly used tool for the measurement of transcript abundance. Currently, ArrayExpress and Gene Expression Omnibus, the two major online repositories of transcriptome data, each contain nearly a million DNA microarray datasets [2,3]. Despite their widespread use, however, concerns have been raised regarding the reproducibility of DNA microarray experiments across microarray platforms, across laboratories and even within the same laboratory [4–9].

RNA sequencing (RNA-seq) has recently become popular as an alternative method for measuring transcript abundance [10]. Like the DNA microarray, RNA-seq is high-throughput, suggesting that it should be possible to use data from one technique to validate the other. Previous studies have examined the level of agreement between DNA microarray data and RNA-seq data, among them [11–17]. Some of these studies indicate a strong correlation between the two techniques, while others demonstrate a substantial discordance, indicating that there are problems either in the techniques themselves or in the manner by which the datasets are compared. Since there is currently no consensus on the concordance between RNA-seq data and DNA microarray data, it is worthwhile to continue to investigate this issue.

While several previous studies have compared RNA-seq data to DNA microarray data [11–17], to our knowledge, only one (by Marioni *et al.* [12]) used quantitative reverse-transcription PCR (qRT-PCR) as an independent validation technique. Further, Marioni *et al.* [12] performed qRT-PCR on only a handful of genes. In this study, we compared transcript abundances in human foreskin fibroblasts that were in one of two states—proliferating (‘PRO’) or quiescent (‘QUI’)—using both DNA microarrays (two-channel OpArray microarrays with approx. 70 bp probes) and RNA-seq (mRNA paired-end Illumina-based sequencing), and then used qRT-PCR to perform an independent measure of transcript abundance for 76 genes. The use of normal human fibroblasts provides a simple system of homogeneous cell populations to avoid ‘noise’ that can mask transcript profiles in more complicated, less homogeneous systems, such as whole tissues. Specifically, we characterized the level of reproducibility of the RNA-seq data, the level of reproducibility of the microarray data, the correlations between the two techniques and the level of agreement of each technique with the qRT-PCR data. Measurements from different RNA-seq reactions applied to cells in the same state were highly consistent with one another, while the microarrays exhibited variable internal reproducibility. The concordance between the RNA-seq data and the individual microarrays was low, while a greater concordance was observed between the RNA-seq data and the geometric mean of the microarrays. The qRT-PCR data were more consistent with the RNA-seq data than with the microarray data. The findings from this study highlight the importance of validating any high-throughput technique to ensure confidence in the biological validity of the data.

## 2. Results and discussion

### 2.1 Reproducibility of DNA microarray data

In order to determine the concordance between transcript abundances as measured by RNA-seq and by DNA microarrays, two RNA-seq reactions and four two-channel DNA microarray assays were performed. We first determined the level of internal reproducibility of the microarray data. Labelled cDNA libraries prepared from paired proliferative and quiescent cells were hybridized to each of four microarrays (OpArray, see Material and methods), with biological replicates used for each microarray. The four microarrays were labelled QP1, QP2, QP3 and QP4. ‘Dye-swaps’ were performed for arrays QP2 and QP4 to ensure that there were no biases in the labelling protocol. Analysis of raw datasets was performed using the online microarray database software BioArray Software Environment (BASE) [18], with which cross-channel correction and LOWESS normalization were performed.

Each microarray contained 35 355 probes, each approximately 70 bp in length. Correlations between probe intensity values (the intensity values for PRO in the first microarray versus the intensity values for PRO in the second microarray, and similarly for QUI) and fold change (FC) values (QUI/PRO) were determined for all (42)=6 pairs of microarrays. Three measures of correlation were calculated: Pearson correlation, Pearson correlation between log-transformed values, and Spearman correlation. Correlations ranged from 0.78 to 0.94 for Pearson correlation, 0.78 to 0.94 for Pearson correlation between log-transformed values, and 0.77 to 0.94 for Spearman correlation (electronic supplementary material, table S1). Scatterplots for the comparisons between log-transformed intensity values are shown in the electronic supplementary material, figures S1–S12.

**Figure 1.**
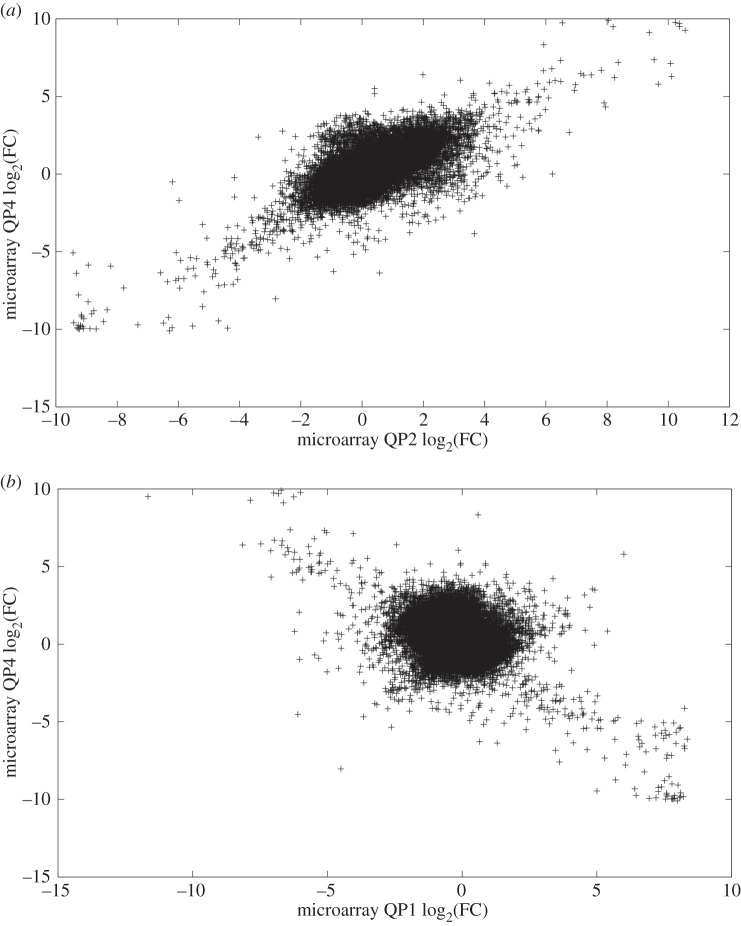
Differing reproducibility of microarray FC values. The log-transformed FC values from some pairs of microarrays were consistent with one another, while negative correlations were observed for other pairs. Panel (*a*) shows the relationship between the log-transformed FC values from microarray QP2 and those from microarray QP4, which exhibited a moderate to strong correlation (*r*=0.74). By contrast, panel (*b*) shows the relationship between the log-transformed FC values from microarray QP1 and those from microarray QP4, which had a negative correlation (*r*=−0.41).

Relative to the correlations between intensity values, the Pearson correlations between FC values were generally lower, ranging from −0.01 to 0.71 (table 1). This was expected given that the intensity values for PRO or QUI represent just a single random variable, whereas FC is a function of two random variables and thus should have greater variance. The Pearson correlations after log-transforming the FC values were highly variable, as were the Spearman correlations (table 1). Both correlation measures were positive between microarrays QP1 and QP3 and between QP2 and QP4, but were negative between all other pairs of arrays. For example, a positive relationship was observed between microarrays QP2 and QP4 (figure 1*a*), while microarrays QP1 and QP4 exhibited a negative relationship (figure 1*b*). These negative correlations were unexpected; however, they might arise from the method used to label specific cDNA libraries. Each library was constructed to bind specific tags called dendrimers (see the Material and methods section for details) following hybridization of the cDNA to the arrays. Given the complex nature of dendrimer binding, it is possible that a number of spots on the arrays are giving values consistently with one dye regardless of the dye swaps for the samples and therefore disrupting the array correlations. For the majority of probes, there appeared to be little relationship between the log-transformed FC values between QP1 and QP4 (the ‘cloud’ in the middle of figure 1*b*); however, probes with very high FC values in one microarray tended to have very low FC values in the other (figure 1*b*), resulting in a negative Pearson correlation. Scatterplots comparing FC values for the remaining pairs of microarrays are given (electronic supplementary material, figures S13–S16).

**Table 1.** Differing reproducibility of microarray FC values. (Correlations between FC values (QUI/PRO) are shown for each pair of microarrays. The values in the upper diagonal contain the Pearson correlations, while those in the lower diagonal contain the Spearman correlations. Values not in parentheses represent correlations between untransformed FC values, while those in parentheses represent correlations between log-transformed FC values. As log transformation does not change the rank order, only one number is shown for the Spearman correlation for each pair. Correlations varied substantially depending on the pair of microarrays and the correlation metric used, ranging from −0.55 to 0.74.)

	QP1	QP2	QP3	QP4
QP1	—	0.00 (−0.46)	0.68 (0.70)	−0.01 (−0.41)
QP2	−0.34	—	−0.01 (−0.55)	0.71 (0.74)
QP3	0.65	−0.43	—	−0.01 (−0.44)
QP4	−0.30	0.68	−0.33	—

### 2.2 Reproducibility of RNA-sequencing data

We previously performed comparative transcriptome analysis of RNA-seq datasets to identify genes that had changed expression more than or equal to fivefold as fibroblasts were induced from proliferative growth to quiescence using serum starvation [19]. Our analyses demonstrated that 751 genes (not probes) changed expression (428 increased and 323 decreased), and that these genes could be mapped to specific biological pathways, including cell cycle control and mitosis, as well as the complement and coagulation cascade. In this study, we re-analysed this RNA-seq data using several criteria to allow us to perform our comparisons with the microarray datasets. Reads were mapped against the GRCh37 reference genome from Ensembl [20] using TopHat 2 [21]. Using SeqMonk (http://www.bioinformatics.babraham.ac.uk/projects/seqmonk), 149 135 probes were generated based on Ensembl-annotated mRNA transcripts, and read counts were normalized using the widely used reads per kilobase of gene per million reads (RPKM) method [22–29]. Electronic supplementary material, figure S17, illustrates the graphical output of SeqMonk for three genes. The two proliferative RNA-seq replicates were labelled PRO1 and PRO2, while the two quiescent replicates were labelled QUI1 and QUI2.

**Figure 2.**
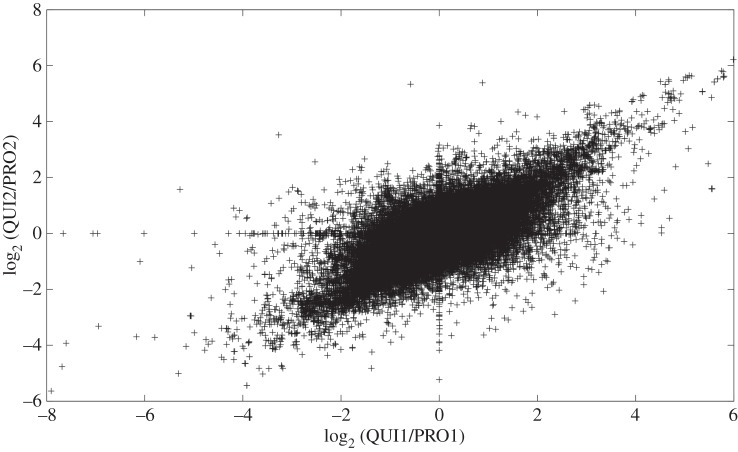
Moderate reproducibility of RNA-seq FC values. The scatterplot shows that there was a moderate to strong linear relationship between the log-transformed FC values for QUI1/PRO1 and those for QUI2/PRO2 (*r*=0.70).

The concordance between the RNA-seq replicates was determined both in terms of normalized read counts (i.e. the number of reads mapped to a given probe for PRO1 versus the number of reads mapped to the same probe for PRO2, and similarly for QUI) and in terms of FC values (i.e. QUI1/PRO1 versus QUI2/PRO2). A fixed value of 0.05 was added to the normalized read count for each probe to prevent division by zero when calculating FC values. While the Pearson correlations between read counts were fairly low (0.58 for PRO and 0.35 for QUI), the Pearson correlations between log-transformed read counts, as well as the Spearman correlations, were similar to or higher than those of the microarray data, ranging from 0.93 to 0.94 (table 2). These correlations indicate that the relationships between the read counts deviated somewhat from linearity, but were close to monotonic. Scatterplots demonstrating the relationship between log-transformed values (electronic supplementary material, figures S18 and S19) confirmed this finding. Similar to the microarray data, where correlations between FC values were lower than between intensity values, the RNA-seq data exhibited lower correlations between FC values than between read counts (table 2 and figure 2).

**Table 2.** High reproducibility of RNA-seq read counts, and moderate reproducibility of RNA-seq FC values. (The correlations between read counts (PRO1 versus PRO2 and QUI1 versus QUI2) and FC values (*QUI*1/*PRO*1 versus *QUI*2/*PRO*2) are shown. Except for the Pearson correlations between non-log-transformed values, correlations between read counts were similar in magnitude to the correlations observed between microarray intensity values (electronic supplementary material, table S1). Correlations between FC values were close to those observed in the most highly correlated pairs of microarrays.)

	read counts		FC values
correlation	QUI	PRO	QUI/PRO
Pearson	0.58	0.35	0.77
Pearson (log)	0.94	0.94	0.70
Spearman	0.93	0.93	0.59

### 2.3 Concordance between RNA-sequencing data and microarray data

In order to compare the RNA-seq data to the microarray data, the correspondence between the microarray probes and the RNA-seq probes had to be ascertained. Both a sequence-based method and a method based on Ensembl transcript IDs were used in order to generate a mapping of microarray probes to RNA-seq probes. Of the 35 355 microarray probes, 29 007 (82%) mapped to an RNA-seq probe via at least one of the two methods. The remaining microarray probes, as well as the RNA-seq probes that did not have a matching microarray probe, were not used in comparing the microarray data to the RNA-seq data. Some RNA-seq probes were mapped to by more than one microarray probe; the total number of unique RNA-seq probes used was 22 041 (14.8% of the total number of RNA-seq probes). Note that because the RNA-seq probes overlap in the genome, it is not the case that 85.2% of the RNA-seq data are being thrown away in the comparison to the microarray data; see the electronic supplementary material, Discussion, for more details.

To simplify the comparisons, the reads from the two PRO RNA-seq replicates were combined, as were the reads for the two QUI RNA-seq replicates. This was justified by the high reproducibility of the RNA-seq biological replicates (table 2). These combined datasets were then compared to each individual microarray (i.e. the microarray data were not combined except as indicated below). Two types of comparisons were performed: first, normalized read counts from the RNA-seq data were compared to normalized intensity values from the microarrays; second, FC values from the RNA-seq data were compared to FC values from the microarrays. For the second comparison, correlations were also calculated between the RNA-seq FC values and the geometric mean of the four microarray FC values. The correlations between RNA-seq read counts and microarray intensity values for both PRO and QUI ranged from 0.18 to 0.22 for Pearson correlation, 0.32 to 0.41 for Pearson correlation between log-transformed values, and 0.29 to 0.40 for Spearman correlation (table 3). The level of concordance between RNA-seq read counts and microarray intensity values is illustrated by scatterplots for each comparison (electronic supplementary material, figures S20–S27).

**Table 3.** Low concordance between RNA-seq data and DNA microarray data. (For each cell state (PRO and QUI), reads from the two RNA-seq replicates were pooled to give a single read count for each probe. Concordance was determined using both correlation between reads counts (for the RNA-seq data) and intensity values (for the microarray data), and between FC values (QUI/PRO). Correlations between read counts and intensity values were low, ranging from 0.18 to 0.41, as were correlations between FC values, which ranged from 0.02 to 0.23. ‘All’ represents the geometric mean of the FC values of the four microarrays. The correlations between the RNA-seq data and the mean of the four microarrays was better than between the RNA-seq data and any of the individual microarrays.)

	PRO read count versus intensity				QUI read count versus intensity				FC (QUI/PRO)				
correlation	QP1	QP2	QP3	QP4	QP1	QP2	QP3	QP4	QP1	QP2	QP3	QP4	all
Pearson	0.22	0.18	0.20	0.21	0.20	0.19	0.20	0.20	0.04	0.07	0.03	0.02	0.42
Pearson (log)	0.33	0.33	0.40	0.37	0.32	0.32	0.41	0.35	0.23	0.18	0.18	0.17	0.42
Spearman	0.30	0.30	0.38	0.33	0.29	0.29	0.40	0.33	0.21	0.18	0.17	0.16	0.34

**Figure 3.**
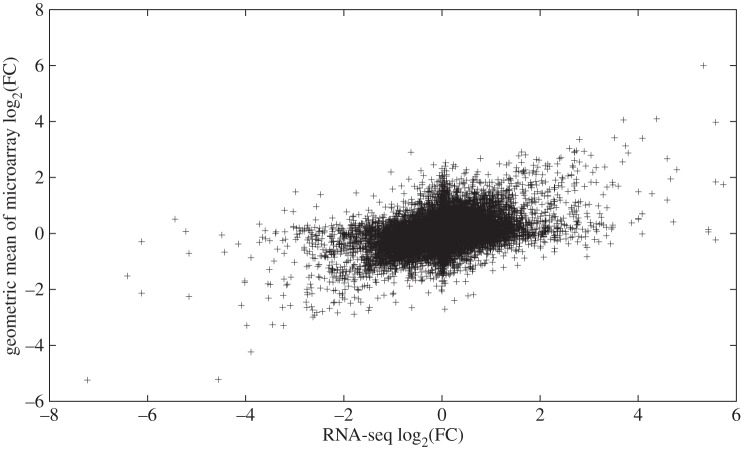
Moderate concordance between the log-transformed RNA-seq FC values and the log-transformed geometric mean of the microarray FC values. The scatterplot shows that there was a moderate linear relationship between these two variables (*r*=0.42).

In §2.1, we reported that the inter-array correlations between some pairs of microarrays were low. In response to this, we compared the microarray data to the RNA-seq data in two different ways: individually, as well as collectively. Comparing each microarray individually to the RNA-seq data has the advantage that the degree of concordance can be ascertained without inter-array variation being a contributing factor. By contrast, comparing the combined microarray data to the RNA-seq data has the advantage that it is more consistent with how experiments are typically done. More specifically, biological inferences are almost never drawn from the results of just a single microarray; it would be far more common to perform several replicates and then combine their results (e.g. by using the geometric mean of the FC values for a given probe). In this study, the correspondence of the FC values between RNA-seq and the individual microarrays was very low, ranging from 0.02 to 0.07 for the Pearson correlation, 0.17 to 0.23 for the Pearson correlation between log-transformed values, and 0.16 to 0.21 for the Spearman correlation (table 3 and electronic supplementary material, figures S28–S31). Thus, none of the individual microarrays agreed with the RNA-seq data. When the data from the microarrays in this study were combined, the concordance between the RNA-seq data and the microarray data was much better than between the RNA-seq data and any of the individual microarrays (figure 3). Specifically, the values of the three correlation measures were 0.42, 0.42 and 0.34, respectively (table 3). Thus, in practical terms, we would characterize the RNA-seq data and the microarray data as having a moderate degree of agreement. Since we observed negative correlations involving two of the arrays (§2.1), and although we were confident that our experimental procedure was performed correctly, we considered the possibility that an error occurred with the dye-swap. We therefore artificially swapped the dyes on these arrays and re-performed our analyses. In the results, the arrays were more in agreement with each other, although this generated more discordance between the array results and the RNA-seq results (data not shown), demonstrating that the negative correlations are not responsible for the disagreement between the two technologies.

The concordance between the two methods was also evaluated by examining the level of overlap of genes that had the greatest differences in transcript abundance according to each technique. Specifically, lists of the 10, 50, 100, 500 or 1000 probes with the highest FC values were determined for each microarray (and also the geometric mean of the four microarrays), as well as for the combined RNA-seq replicates. For each list size, the RNA-seq list was compared separately to the list for each microarray. Using the empirical statistical test described in Material and methods, a *p*-value was determined which indicates the likelihood that the level of overlap between a given pair of lists was greater than would be expected at random. For lists of size 10, there was no overlap between the RNA-seq data and microarrays QP1, QP3 and QP4, while there was one probe in common between the RNA-seq data and microarray QP2 (table 4). Two probes were in common between the mean microarray list and the RNA-seq list. For lists of size 100, 500 and 1000, all microarrays had more overlap with the RNA-seq data than would be expected at random. As with the correlations reported above, the degree of concordance was greater between the RNA-seq data and the geometric mean of the four microarrays than between the RNA-seq data and any of the individual microarrays (table 4, last row). For instance, for lists of size 50, 12 probes were in common between the RNA-seq data and the combined microarray data, whereas for the same list size, the greatest number of probes in common between the RNA-seq data and one of the individual microarrays was 3. A list of the probes in common between the combined microarray data and the RNA-seq data for each list size is given in electronic supplementary material, table S2. In addition to probes having the greatest increase in transcript abundance in QUI relative to PRO, the same data were also determined for probes having the greatest decrease in transcript abundance (that is, the smallest FC values). The results for both analyses were very similar (electronic supplementary material, tables S3 and S4).

**Table 4.** Moderate overlap between the probes with the highest FC values in the RNA-seq data and those with the highest FC values in the DNA microarray data. (*k* represents the size of a given list (the 10, 50, 100, 500 or 1000 probes with the highest FC values), while *n* represents the number of probes in common between a list from the RNA-seq data and the corresponding list from the DNA microarray. The *p*-value represents the proportion of 10 000 random trials that had an equal or greater level of overlap than that actually observed. Thus, if none of the random trials had a greater level of overlap, then the *p*-value is 0. More overlapping probes than would be expected by chance were observed for all microarrays for *k*=100, 500 and 1000, while some arrays had statistically significant *p*-values for *k*=10 and *k*=50. ‘All’ represents the geometric mean of the FC values of the four microarrays.)

	*k*=10		*k*=50		*k*=100		*k*=500		*k*=1000	
	*n*	*p*-value	*n*	*p*-value	*n*	*p*-value	*n*	*p*-value	*n*	*p*-value
QP1	0	1	1	0.08	9	0	95	0	190	0
QP2	1	0.004	2	0.003	4	0.0003	54	0	97	0
QP3	0	1	3	0	9	0	87	0	187	0
QP4	0	1	1	0.08	5	0	39	0	85	0
all	2	0	12	0	23	0	131	0	257	0

Finally, of the 29 007 instances in which there was a corresponding probe between the RNA-seq data and the microarray data, the number of microarray probes that had an FC value more than 5 was 225, 785, 256, 975 and 50 for QP1, QP2, QP3, QP4 and the geometric mean of the four arrays, respectively. Of these, the numbers of corresponding RNA-seq probes that also had an FC value more than 5 were 31 (13.8%), 30 (3.8%), 26 (10.2%), 27 (2.8%) and 27 (54.0%), respectively. Thus, consistent with the results given above, the geometric mean of the four microarrays was far more concordant with the RNA-seq data than the individual microarrays.

The reason for the modest agreement between the RNA-seq data and the microarray data is unclear. Although all samples used in this study were biological replicates, the high internal reproducibility of both the microarray data and the RNA-seq data indicates that biological variability is an unlikely explanation. It is possible that the inability of DNA microarrays to distinguish between splice variants may play a role, as could the difficulty exhibited by DNA microarrays in detecting transcripts with low abundances. It is also possible that there are unforeseen biases that impact the results from RNA-seq.

The degree of concordance between DNA microarray data and RNA-seq data has been examined in several previous studies. For instance, Sultan *et al.* [11] examined differences in transcript abundance between two human cell lines (embryonic kidney cells and B cells) and observed a high correlation (0.88) between the log-transformed FC values derived from the two techniques. Similarly, Marioni *et al.* [12] compared transcript abundance profiles between human liver and kidney cells. The authors computed the correlation in two different ways: between the log-transformed normalized read counts from the RNA-seq data and the normalized intensity values from the microarray, and between the FC values (*liver*/*kidney*) from the RNA-seq data and the FC values from the microarray data. The correlations from both methods were similar, ranging from 0.67 to 0.75. In a review article, Wang *et al.* [13] compared data from a study that examined the yeast transcriptome using DNA microarrays [30] and a study that examined the yeast transcriptome using RNA-seq [10]. The authors found that the correlation was very low (between 0.099 and 0.177) at low transcript abundances (according to RNA-seq), but higher (0.509) at moderate transcript abundances, perhaps reflecting the lower dynamic range of DNA microarrays. Fu *et al.* [14] used three different techniques to examine gene expression in human brain samples: DNA microarrays, RNA-seq and mass spectrometry. Depending on the samples used, the correlation between the RNA-seq data and the microarray data ranged from 0.51 to 0.67. Protein abundances, as quantitated using mass spectrometry, correlated with the abundances of the corresponding mRNAs more tightly for the RNA-seq data than for the microarray data. In yet another comparative study, the transcriptomes of male and female *Drosophila pseudoobscura* were characterized to identify genes whose transcription is sex biased [15]. High correlations were observed when directly comparing microarray intensity values with RNA-seq read counts, although lower correlations were reported when comparing FC values. A strong concordance between DNA microarray data and RNA-seq data was also observed in human T cells [16] and in a rat pain model [17]. A summary of the correlations observed in this study and the studies described above is given in the electronic supplementary material, table S5. The studies described to date vary substantially in the strength of the reported correlations, demonstrating a lack of consistency when evaluating the extent to which the two techniques agree. Here, we report a degree of concordance between RNA-seq data and DNA microarray data that is consistent with, and at the lower end of, those reported thus far.

### 2.4 Consistency between quantitative reverse-transcription PCR data and RNA-sequencing/microarray data

In order to provide measurements via an independent technique, qRT-PCR was used to measure transcript abundance for 76 genes. Each of these genes had a corresponding probe in both the microarray data and the RNA-seq data. Several genes were selected because they had FC values more than 5 in either the RNA-seq data only, the microarray data only, or both. Other genes were selected arbitrarily. Over all 76 genes, the RNA-seq data were more consistent with the qRT-PCR data than were the microarray data (table 5 and electronic supplementary material, figure S32), with correlations of 0.35, 0.56 and 0.56 (Pearson correlation, Pearson correlation of log-transformed values and Spearman correlation, respectively) for the RNA-seq data versus 0.25, 0.48 and 0.44 for the microarray data. The gulf between the RNA-seq data and the microarray data was greater for genes with more than fivefold differential expression according to qRT-PCR (*n*=35). In particular, there were 23 genes for which the log-transformed RNA-seq FC value was closer to the qRT-PCR FC value than was the mean of the log-transformed microarray FC values, versus 12 for the reverse (electronic supplementary material, table S6).

**Table 5.** RNA-seq FC values correlate better with qRT-PCR FC values than do microarray FC values, although not to a statistically significant degree. Correlation coefficients are shown between the qRT-PCR FC values for 76 genes, and the FC values for corresponding probes in each individual microarray or in the combined RNA-seq replicates. ‘All’ represents the geometric mean of the FC values of the four microarrays. For all three correlation measures, the RNA-seq correlation was not significantly different (*p*-value >0.05) from the correlation of any of the microarrays (Fisher's *z*-transformation).

	microarrays					
correlation	QP1	QP2	QP3	QP4	All	RNA-seq
Pearson	0.31	0.15	0.34	0.18	0.25	0.35
Pearson (log)	0.39	0.43	0.38	0.35	0.48	0.56
Spearman	0.39	0.45	0.42	0.34	0.44	0.56

## 3. Conclusion

As more laboratory techniques are developed that generate large volumes of data, assessing the reproducibility of that data becomes ever more important. DNA microarrays are one of the most widely used high-throughput techniques, with around a million datasets deposited in online databases [2,3]. As sequencing costs continue to decrease, the rate by which RNA-seq data are generated will continue to climb, providing an extensive reservoir of information on transcript profiles from a variety of sources. Given that the two technologies are both used to measure transcript abundance, one would expect them to yield consistent results. However, previous studies that have compared RNA-seq data to DNA microarray data have reported widely varying degrees of concordance.

In this study, we measured transcript abundances in proliferative and quiescent fibroblasts using both DNA microarrays and RNA-seq. With some exceptions, replicates within the same technology were reproducible; however, the agreement between them was modest, although better agreement was observed for genes with large differences in transcript abundance between the proliferative and quiescent samples. Better agreement was also achieved when the RNA-seq data was compared against the geometric mean of the four microarrays rather than the individual microarrays. As an independent assessment, qRT-PCR was used to measure transcript abundance for several genes, and it was found that the qRT-PCR data were more consistent with the RNA-seq data than with the microarray data. As such, this study highlights the benefit of evaluating the reproducibility of transcript abundance measurements by using multiple independent techniques. More generally, for any type of biological measurement, this study indicates that the measurement should be made using more than one method when the opportunity exists to do so.

When considering the level of concordance between microarray- and RNA-seq-based transcriptome profiling, one must also consider the question to be addressed. For most biological studies, the question is probably aimed at identifying which genes have the most significant increase or decrease in expression/transcript abundance as a function of the experimental conditions. From this standpoint, our observations between RNA-seq and microarray demonstrated moderate concordance, with those genes identified to change expression in both datasets likely representations of true changes in gene expression. From an informatics perspective, the question may focus on the overall measurement across all genes, not just those that have changed significantly. Our data indicate that the low concordance across the entire dataset may require caution when interpreting data for informatics studies, but that a comparison of both data types will yield useful interpretations when identifying genes that have most significantly changed.

## 4. Material and methods

### 4.1 Cell growth

Normal human foreskin fibroblasts (2DD) [31,32] were grown in Dulbecco's modified Eagle's medium. Proliferative cells (less than passage 15) were cultured in 10% foetal bovine serum (FBS) and seeded at an initial density of 3000 cells cm^−2^. Media were changed every 3–4 days with cells never becoming confluent. Quiescence was induced in 2DD cells by replacing normal growth media with 0.5% FBS containing media for 7 days [31,32].

### 4.2 RNA extraction and cDNA synthesis

More than 10 million cells for both proliferating and quiescent samples were harvested using Trypl Express (Invitrogen). Cells were pelleted by centrifugation and RNA extracted using either Trizol (Invitrogen) or FastRNA Pro Green Kit with FastPrep-24 instrument (MP Biomedicals) according to the manufacturer's instructions. Polyadenylated mRNAs were isolated from the RNA pools using the Oligotex mRNA isolation kit (Qiagen). Five micrograms of RNA from both proliferating and quiescent cells were used in cDNA synthesis reactions in combination with Superscript III (Invitrogen) as per manufacturer's instructions with random hexamers (50 ng per reaction). The final reaction was diluted to a final volume of 200 μl.

### 4.3 Generation of microarray data

Microarray analysis was carried out using Op Human ReadyArray HS1200 (Microarrays Inc.) slides, with the 3DNA Array 900 labelling kit (Genisphere) as previously described [33]. Four microarray assays were performed, each with samples from cDNA isolated from four independent PRO/QUI samples. For two of the microarrays, Cy3 was used for the proliferative cells and Cy5 for the quiescent cells, and vice versa for the other two microarrays.

#### 4.3.1 cDNA synthesis for microarrays

One microgram of RNA was taken in a volume of 5 μl H_2_O. One microlitre of RT primer was added to the RNA, with the correct dendrimer target sequence for labelling of the samples on the microarray. The mixture was heated to 80°C for 5 min to denature RNA secondary structures and placed on ice for 2 min. The following reagents were added to each reaction: 2 μl of first-strand buffer, 1 μl of 0.1 M DTT, 0.5 μl of SUPERase-In (provided with the 3DNA 900 kit), 0.5 μl of dNTP mix (provided with the 3DNA 900 kit) and 0.5 μl of SuperScript III (Invitrogen). The reaction was incubated for 2 h at 42°C. The reaction was then stopped by adding 1 μl of 1 M NaOH/100 mM EDTA solution and incubating at 65°C for 10 min to denature the cDNA/RNA hybrids and degrade the template RNA. The reverse transcription reaction was then neutralized by adding 1.2 μl of 2 M Tris-HCl (pH 7.5). One microlitre of H_2_O was then added to each cDNA sample and the samples mixed. One microlitre of sample was assessed using the Qubit^®^ single-stranded DNA assay on a Qubit 1.0 Fluorometer to verify that a sufficient quantity of cDNA was present.

The appropriate samples were then mixed to form the hybridization mix for the microarray slides. The total amounts (12.7 μl) of each cDNA were mixed with 40 μl of 2× sodium dodecyl sulfate (SDS)-based hybridization buffer and 14.6 μl of H_2_O, to a final volume of 80 μl. The mixture was heated to 80°C for 10 min in order to denature secondary structures, and then cooled to 60°C to prepare it for addition to the slide.

#### 4.3.2 Hybridization

The microarray slides were pre-scanned with the GenPix 5.1 scanner to check for any manufacturing faults. The slides were then pre-hybridized at 65°C for 20 min in a Coplin jar containing 3.5× saline sodium citrate (SSC), 0.1% SDS and 10 mg ml^−1^ bovine serum albumin solution in a volume of 50 ml. The slides were then washed in MilliQ water for 1 min, in isopropanol for 1 min and dried using a Microarray high-speed centrifuge (Arrayit Corporation). The slide was then pre-scanned again with the GenPix 5.1 scanner to verify that it was clean and undamaged.

The microarray slide was then placed into a clean SlideBooster (Advalytix) on a layer of 45 μl AS100 AdvaSon coupling solution (Beckman Coulter), with 60 μl more in the thumb hole at the base of the slide. The wells of the slide booster were each filled with 500 μl of AdvaHum AM102 humidifying solution (Beckman Coulter), and a 24×60 mm LifterSlip was placed on top of the microarray slide. The assembly was pre-warmed to 55°C, and when it reached temperature, the hybridization solution was pipetted underneath the LifterSlip. The microarrays were then hybridized for 16 h.

The slides were then washed in 2× SSC, 0.2% SDS at 55°C for 10 min with orbital rotation of 150 r.p.m., followed by a wash in 2× SSC at room temperature with orbital rotation of 150 r.p.m. for 10 min, followed by a wash in 0.2× SSC at room temperature with orbital rotation of 150 r.p.m. for 10 min. The slides were dried using a Microarray high-speed centrifuge (Arrayit Corporation).

For each slide, 2.5 μl of the Cy3 dendrimer capture reagent was mixed with 2.5 μl of the Cy5 dendrimer capture reagent, with 40 μl of 2× SDS-based hybridization buffer and 35 μl of H_2_O to a final volume of 80 μl to make the second hybridization mix. This was heated at 80°C for 10 min, and then cooled to 55°C to prepare it for addition to the slide. The SlideBooster was assembled as before, and pre-warmed to 50°C. When it was warm, the second hybridization mix was added, and the microarrays were incubated for 4 h. The washing and drying steps described above were then repeated.

### 4.4 Microarray data analysis

Microarray images were imported into BlueFuse v. 3.2 and spots with insufficient signal above background removed from the datasets either manually or by automatic exclusion. Output from BlueFuse was modified into a fused file format and imported into the BioArray Software Environment (BASE) tool [18]. Using BASE, the raw intensity readings from each microarray were subjected to cross-channel correction in order to correct for cross-talk between the fluorophores [34], and the LOWESS method [35] was used to perform within-array normalization of intensity values.

### 4.5 Generation of RNA-sequencing data

Two RNA-seq replicates were used, as recommended by the ENCODE Consortium's Standards, Guidelines and Best Practices for RNA-Seq (http://genome.ucsc.edu/ENCODE/protocols/dataStandards/ENCODE.RNAseq_Standards.V1.0.pdf). RNA was isolated using the FastPrep-24 instrument (MP Biomedicals) according to the manufacturer's instructions. The integrity of the RNA was determined using the Bioanalyzer (Agilent Technologies) with RNA having an RNA integrity number above 9.0 used for further analysis. For sequencing library synthesis, polyadenylated RNAs were purified using oligo dT-beads (Invitrogen) with random hexamers, then used as primers for the cDNA library construction prior to paired-end sequencing. One PRO sample and one QUI sample were sequenced using the Illumina GxII platform at the Centre for the Analysis of Genome Evolution and Function (University of Toronto, Canada). These datasets were denoted PRO1 and QUI1, respectively. Another PRO sample and QUI sample (biological replicates) were sequenced using the same platform at the Babraham Institute (UK), and the corresponding datasets denoted PRO2 and QUI2, respectively. All sequencing reactions resulted in the generation of 50 bp paired-end reads.

### 4.6 RNA-sequencing data analysis

RNA-seq reads were subjected to quality control using the standard Illumina pipeline. Raw sequence reads were mapped against the reference genome (the GRCh37 assembly from Ensembl [20]) using the following command to TopHat 2 [21]: --bowtie1 -p 8 -r 20 --solexa-quals --coverage-search --microexon-search --library-type fr-unstranded. No trimming of reads was performed prior to mapping. The BAM files produced by TopHat 2 were then imported into SeqMonk (http://www.bioinformatics.babraham.ac.uk/projects/seqmonk). The feature probe generator function in SeqMonk was used to generate probes based on mRNA annotations from Ensembl. The number of reads that mapped to each probe was then quantitated, and normalized using the widely used RPKM method [22–29]. A constant value of 0.05 was added to each value in order to prevent cases of division by zero when calculating FC values. A detailed list of the parameter values selected for data importation, probe generation, and read count normalization and quantitation is available in the electronic supplementary material, table S7. The method used to select probes that correspond to those on the microarrays is given in a following section.

### 4.7 Quantitative reverse-transcription PCR

Templates from cDNA synthesis reactions were diluted 1 : 100 in nuclease-free water. Ten microlitre reactions were set up using 5 μl IQ 2X Master Mix (BioRad), 1 μl template, 1.5 μL H_2_O and 2.5 μL 3 μM forward and reverse primers. All reactions for each gene were run in triplicate and were conducted using the RotorGene qPCR machine (Qiagen).

### 4.8 Correlation calculations

Correlations between variables were determined in three different ways: Pearson correlation between untransformed values, Pearson correlation between log-transformed values (i.e. the base-2 logarithm was taken of each variable before determining the Pearson correlation), and the Spearman rank correlation. The statistical significance of differences between correlations was calculated by applying Fisher's *z*-transformation to the correlation coefficients. Log-transformation of variables was done after other transformations (i.e. RPKM for the RNA-seq data and LOWESS normalization for the microarray data).

### 4.9 Reproducibility of DNA microarray data

The consistency among the four microarrays was evaluated by comparing both normalized intensity values and FC values among pairs of microarrays. Specifically, for each of the (42)=6 possible pair of microarrays, the correlation between the intensity values for the PRO channel in the first microarray and the intensity values for the PRO channel in the second microarray was determined, and similarly for the QUI channels. In addition, FC values (QUI/PRO) were compared in each possible pair of microarrays. All microarray probes were used, not just those for which a corresponding RNA-seq probe was identified.

### 4.10 Reproducibility of RNA-sequencing data

The reproducibility of the RNA-seq data was evaluated by comparing both normalized read counts and FC values. In the first method, the read counts from PRO1 were compared to those from PRO2, and read counts from QUI1 were compared to those from QUI2. The second method involved finding the correlation between QUI1/PRO1 and QUI2/PRO2. All RNA-seq probes were used, not just those for which a corresponding microarray probe was identified.

### 4.11 Mapping of RNA-sequencing probes to microarray probes

In order to determine the correspondence between the (real) probes on the DNA microarrays and the (virtual) probes generated by SeqMonk, two different methods were used: a method based on probe sequences and a method based on Ensembl transcript IDs.

The sequence-based mapping method was performed as follows. Associated with each microarray probe *M*_*i*_ was the sequence *M*^S^_*i*_ of that probe, as well as the chromosome *M*^C^_*i*_ on which that sequence is found. The record associated with each probe *R*_*j*_ generated by SeqMonk contained the chromosome *R*^C^_*j*_ on which that probe is found, as well as the start and end position of the probe on that chromosome. Let *R*^S^_*j*_ denote the sequence bounded by those chromosome locations. For each microarray probe *M*_*i*_, it was determined whether there was a SeqMonk probe *R*_*j*_ on chromosome *M*^C^_*i*_ for which *M*^S^_*i*_ was a subsequence of *R*^S^_*j*_. If there did exist such a probe, then *M*_*i*_ was considered to correspond to *R*_*j*_.

If there did not exist a probe *R*_*j*_ for which *M*^S^_*i*_ was a subsequence of *R*^S^_*j*_, then a second mapping method was attempted based on Ensembl transcript IDs. Each SeqMonk probe had exactly one Ensembl ID *R*^E^_*j*_ associated with it. Some microarray probes had exactly one associated Ensembl ID; others had more than one or none at all. Let *M*^E^_*i*_ denote the (possibly empty) set of Ensembl IDs associated with microarray probe *M*_*i*_. For each *e*∈*M*^E^_*i*_, it was determined whether there was a SeqMonk probe *R*_*j*_ such that *e*=*R*^E^_*j*_. If there was, then *M*_*i*_ was considered to correspond to *R*_*j*_ (and no further elements of *M*^E^_*i*_ were examined). For microarray probes having *M*^E^_*i*_=∅, this mapping method could not be performed.

In many cases, a given microarray probe mapped to more than one RNA-seq probe (typically representing splice variants of the same gene) based on sequence. In such cases, if one of the RNA-seq probes was also a match based on Ensembl IDs, then that RNA-seq probe was selected. If not, then one of the identified RNA-seq probes was arbitrarily selected to correspond to that microarray probe.

If no mapping could be found for a given microarray probe via either sequence or Ensembl IDs, then it was not included in the comparison between the microarray data and the RNA-seq data.

### 4.12 Concordance between RNA-sequencing data and microarray data

Prior to comparing the RNA-seq data and the microarray data, the reads from the two RNA-seq replicates for each cell state were combined, giving a single read count for PRO and a single read count for QUI for each probe. These combined data were then compared to each individual DNA microarray. All comparisons were performed using only microarray probes for which a corresponding RNA-seq probe was found (and vice versa).

The RNA-seq data were compared to the microarray data in three ways. First, the RNA-seq read counts for PRO were compared to the PRO intensity values from each microarray, and similarly for QUI. Second, the FC values (QUI/PRO) from the RNA-seq data were compared to the FC values from each microarray (and also the geometric mean of the four microarrays). Third, the degree of overlap between the two techniques was determined in terms of the probes having the greatest FC values (QUI/PRO). Let *S* represent the set of all probes for which a correspondence was found between the microarray data and the RNA-seq data. The *k*=10 probes in *S* with the highest FC values according to the RNA-seq data were identified, as were the *k*=10 probes from *S* having the highest FC values in a given DNA microarray. The number of probes *n* that were in both lists was then ascertained. To determine whether *n* was greater than would be expected by chance, an empirical statistical distribution was calculated by performing 10 000 random trials. In each trial, *k* probes were randomly selected without replacement from *S*. The selected probes were then placed back into *S*, and *k* additional probes were selected without replacement. The number of probes *p* found in both lists was recorded. The associated *p*-value was equal to the number of random trials for which *p*≥*n*. The above procedure was repeated for *k*=50, 100, 500 and 1000.

### 4.13 Consistency between quantitative reverse-transcription PCR data and RNA-sequencing/microarray data

qRT-PCR reactions were conducted using both PRO and QUI samples for 76 genes. Each of these genes had a corresponding probe in both the RNA-seq data and the microarray data. Some genes were selected arbitrarily, while others were selected because they were upregulated in either the RNA-seq data, the microarray data or both. For both PRO and QUI, the cycle threshold *C*_*T*_ was calculated for each gene, as well as for four normalizing genes (*PRDX5*, *EFEMP2*, *FAU* and *FKBP10*). The FC in transcript abundance was calculated relative to each normalizing gene using the ΔΔ*C*_*T*_ calculation [36]. The average FC value among the four normalizing genes was used for comparing to the RNA-seq data and microarray data. If there were multiple microarray probes for a given gene, then the geometric mean of the FC values of those probes was used.

## Supplementary Material

Supplementary discussion (supplementary_discussion.pdf) - Contains the supplementary discussion references in the paper.

## Supplementary Material

Supplementary figures and tables (supplementary_figures_and_tables.pdf) - contains all of the supplementary figures and tables referenced in the paper.

## Data accessibility

The data used for this study have been deposited in Gene Expression Omnibus [3] under the accession nos. GSE66780 (microarray data) and GSE65145 (RNA-seq data).
