# Supplementary material for: Concordance between RNA-sequencing data and DNA microarray data in transcriptome analysis of proliferative and quiescent fibroblasts
Source: R Soc Open Sci. 2015 Sep 30;2(9):150402. doi: 10.1098/rsos.150402 (PMC4593695; doi:10.1098/rsos.150402)

Concordance between RNA-seq data and DNA microarray data in transcriptome analysis of proliferative and quiescent fibroblasts

Explanation of RNA-seq data usage

In the main paper, it was reported that 29,007 of the 35,355 microarray probes mapped to 22,041 unique RNA-seq probes, which constitutes 14.8% of the 149,355 probes generated by SeqMonk. Initially, this could be interpreted as 85.2% of the RNA-seq data being discarded. However, there are two reasons that this is not the case:

- many of the probes generated by SeqMonk represent different splice variants and thus overlap in the genome; and
- each RNA-seq read may map to several probes.

For example, suppose that SeqMonk generates three overlapping probes labeled RP1, RP2, and RP3, as shown in the diagram below. Further, suppose that a particular microarray probe, labeled MP, falls within a region of the genome that overlaps all three of those probes. In our procedure for mapping microarray probes to RNA-seq probes, each microarray probe is only allowed to map to one RNA-seq probe. Suppose that RP2 is selected as the matching microarray probe for MP. When read counts are quantitated by SeqMonk, most of the reads that map to RP1 and RP3 will also map to RP2 (green-colored reads in the diagram below), while a smaller portion will not (red-colored reads). Thus, the information provided by most of the reads is still being used, and as a result, only a small amount of RNA-seq data is being ignored by not including RP1 and RP3 in the comparison.

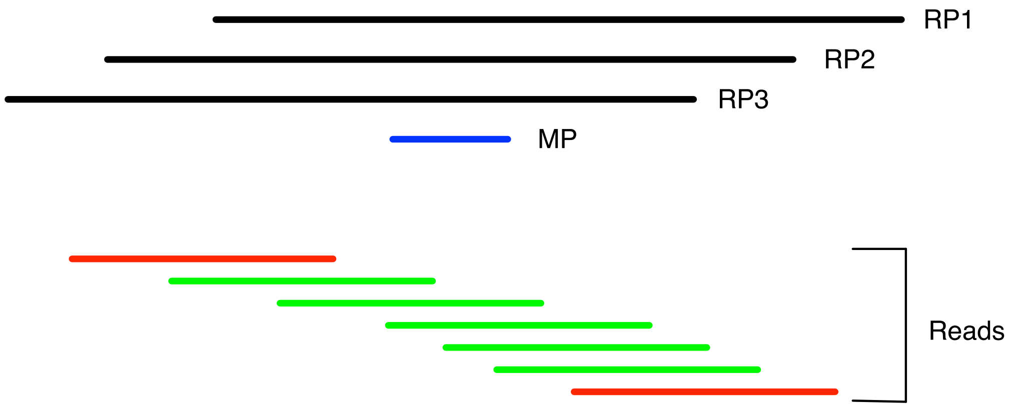

Supplement: Supplementary discussion (supplementary_discussion.pdf) - Contains the supplementary discussion references in the paper. [file rsos150402supp1.pdf]
