# Supplementary material for: Concordance between RNA-sequencing data and DNA microarray data in transcriptome analysis of proliferative and quiescent fibroblasts
Source: R Soc Open Sci. 2015 Sep 30;2(9):150402. doi: 10.1098/rsos.150402 (PMC4593695; doi:10.1098/rsos.150402)
Supplement: Supplementary figures and tables (supplementary_figures_and_tables.pdf) - contains all of the supplementary figures and tables referenced in the paper. [file rsos150402supp2.pdf]

Concordance between RNA-seq data and DNA microarray data in transcriptome analysis of proliferative and quiescent fibroblasts

Supplementary figures and tables

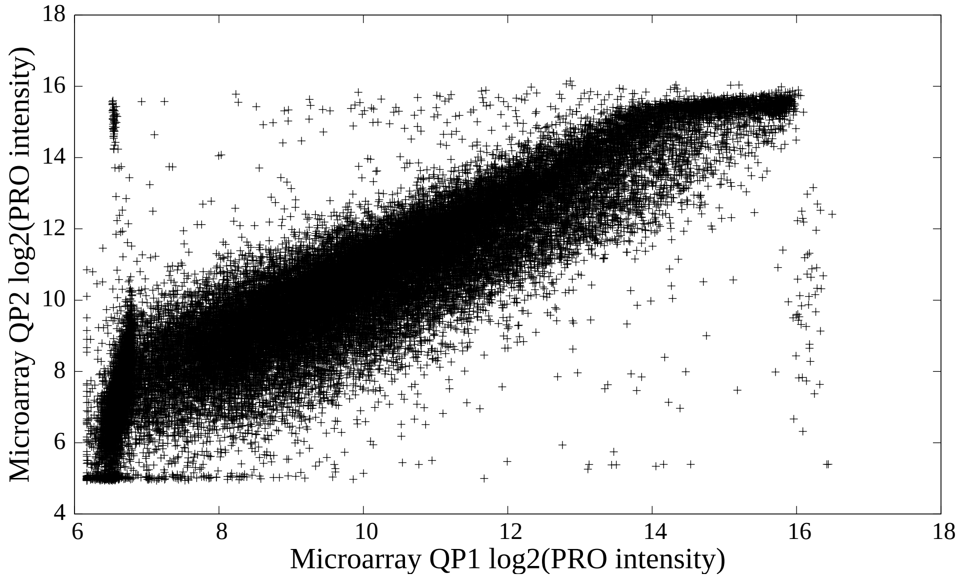

Supplementary Figure S1: The log-transformed PRO intensity values from microarray QP1 and microarray QP2 had a strong correlation ($r = 0.89$).

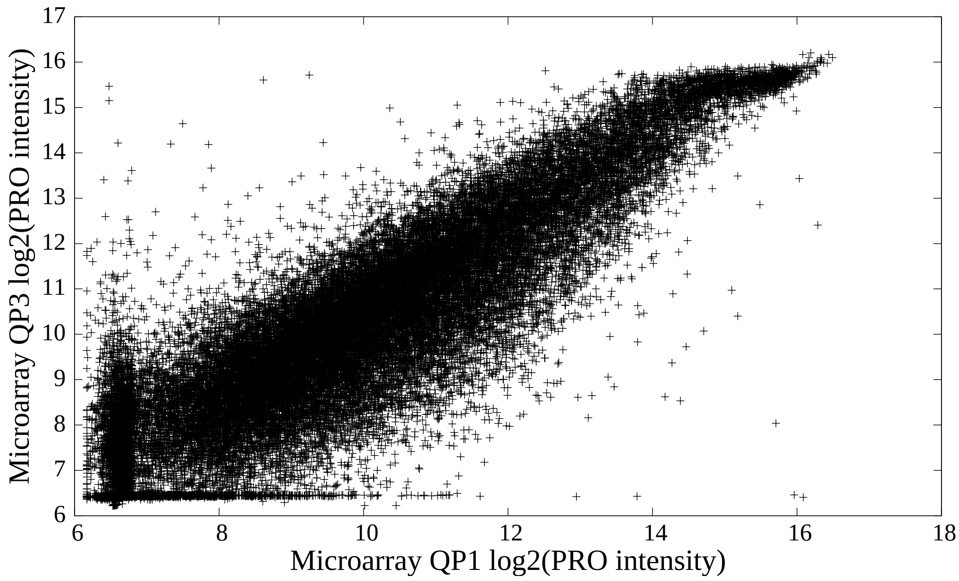

Supplementary Figure S2: The log-transformed PRO intensity values from microarray QP1 and microarray QP3 had a strong correlation ($r = 0.89$).

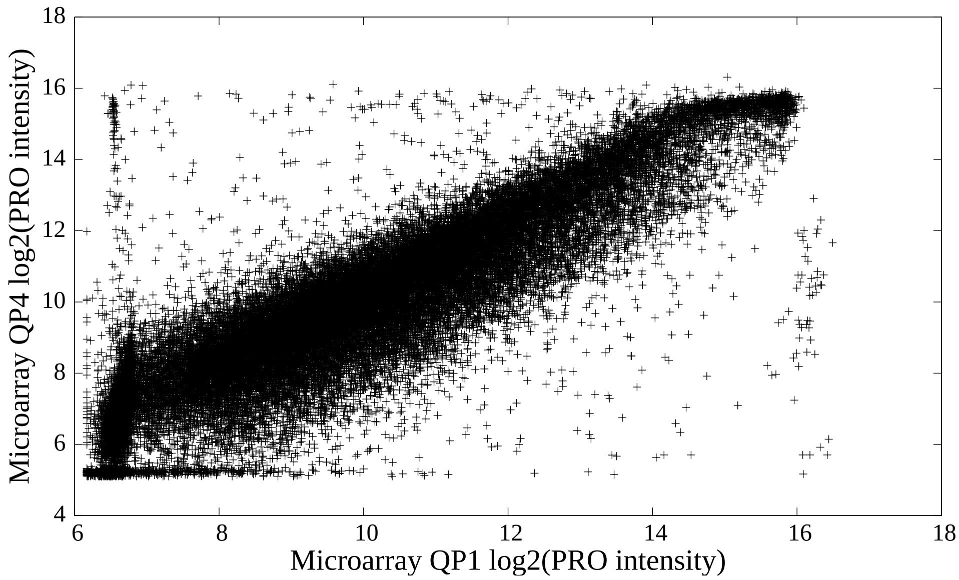

Supplementary Figure S3: The log-transformed PRO intensity values from microarray QP1 and microarray QP4 had a strong correlation ($r = 0.87$).

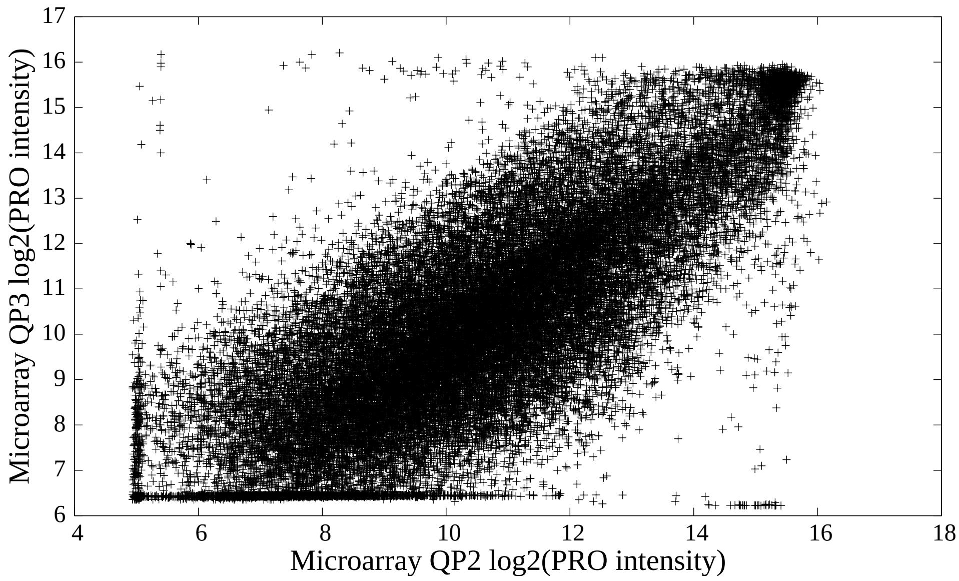

Supplementary Figure S4: The log-transformed PRO intensity values from microarray QP2 and microarray QP3 had a moderately strong correlation ($r = 0.78$).

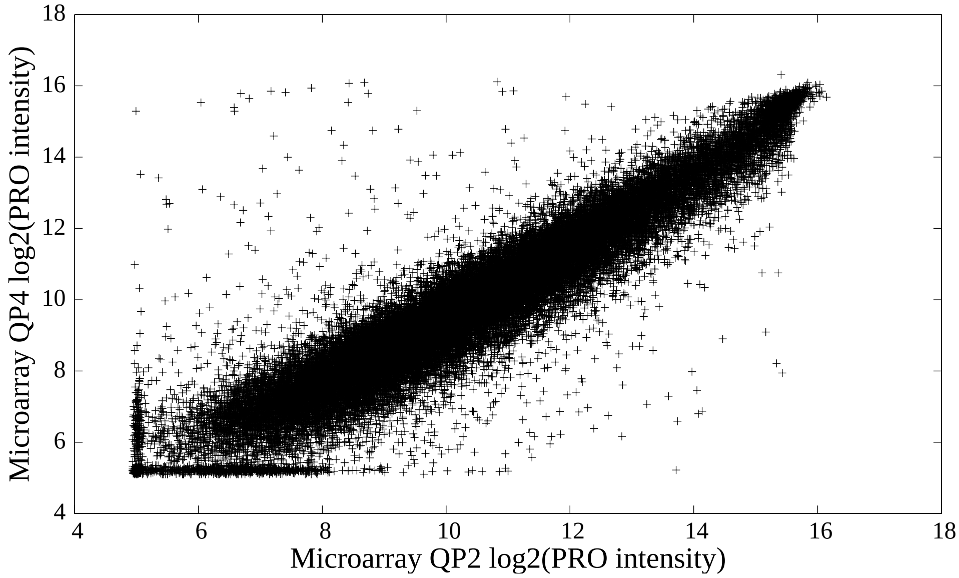

Supplementary Figure S5: The log-transformed PRO intensity values from microarray QP2 and microarray QP4 had a strong correlation ($r = 0.94$).

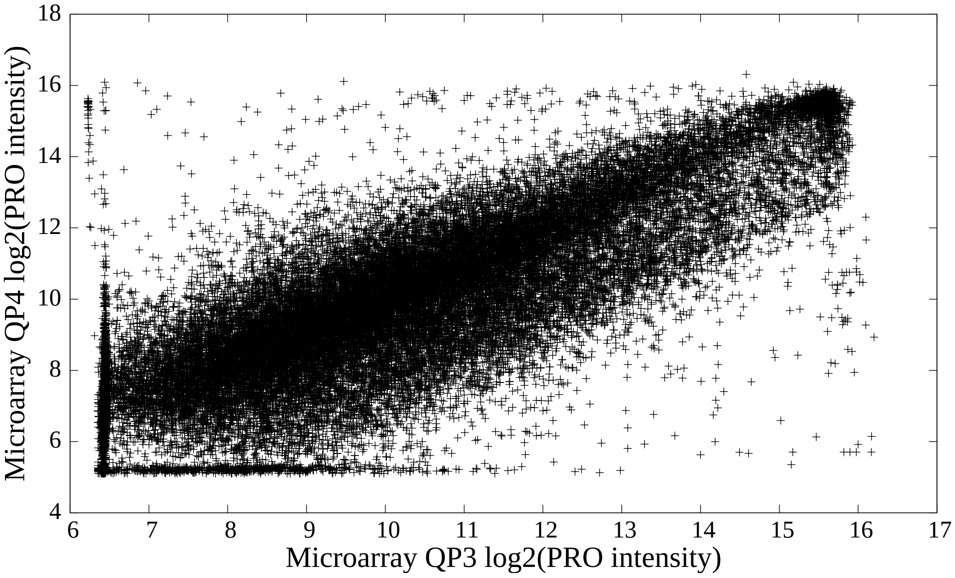

Supplementary Figure S6: The log-transformed PRO intensity values from microarray QP3 and microarray QP4 had a strong correlation ($r = 0.80$).

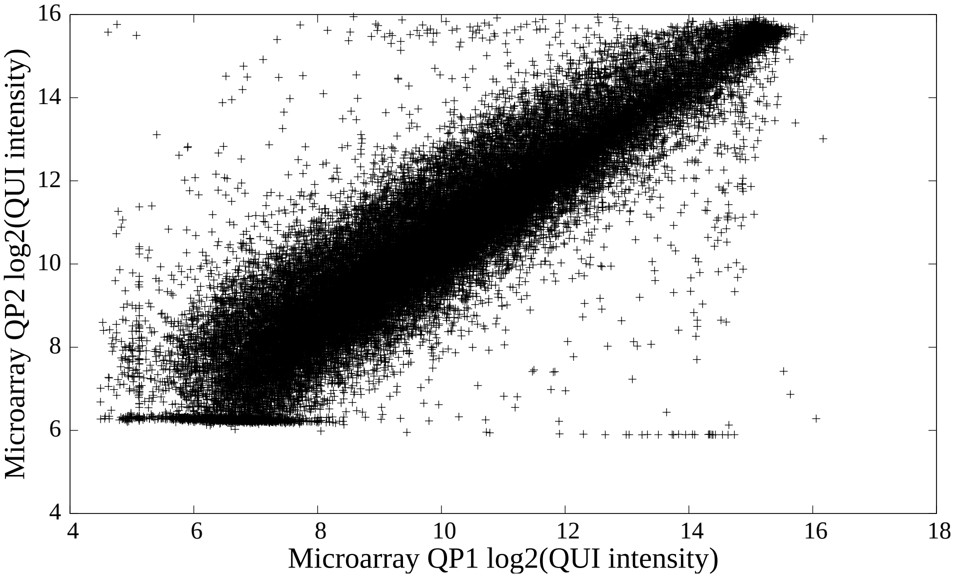

Supplementary Figure S7: The log-transformed QUI intensity values from microarray QP1 and microarray QP2 had a strong correlation ($r = 0.91$).

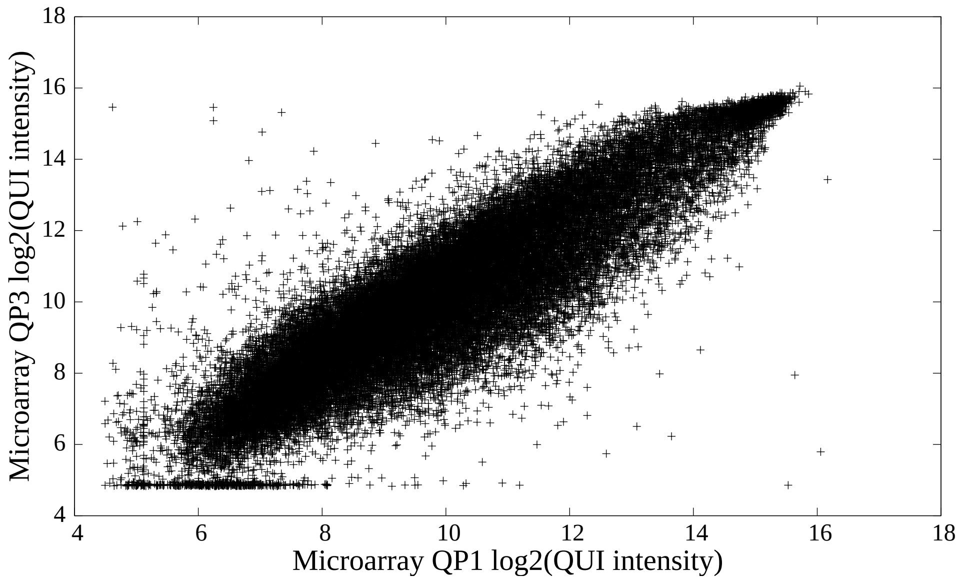

Supplementary Figure S8: The log-transformed QUI intensity values from microarray QP1 and microarray QP3 had a strong correlation ($r = 0.90$).

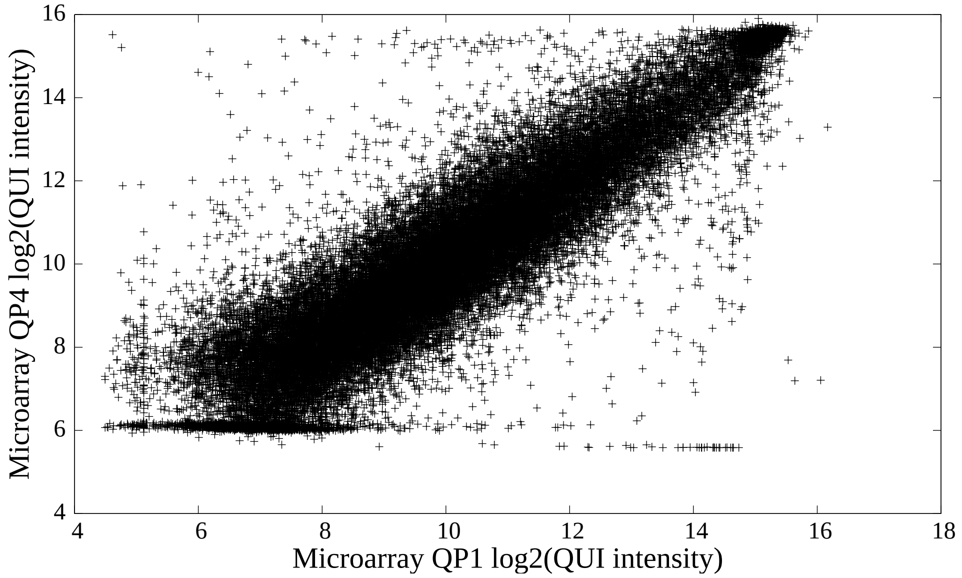

Supplementary Figure S9: The log-transformed QUI intensity values from microarray QP1 and microarray QP4 had a strong correlation ($r = 0.89$).

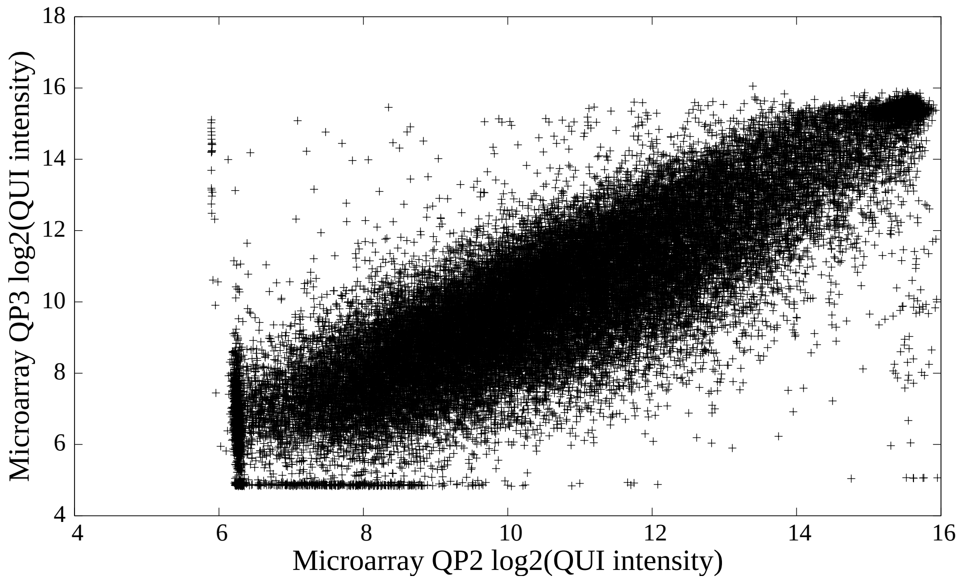

Supplementary Figure S10: The log-transformed QUI intensity values from microarray QP2 and microarray QP3 had a strong correlation ($r = 0.85$).

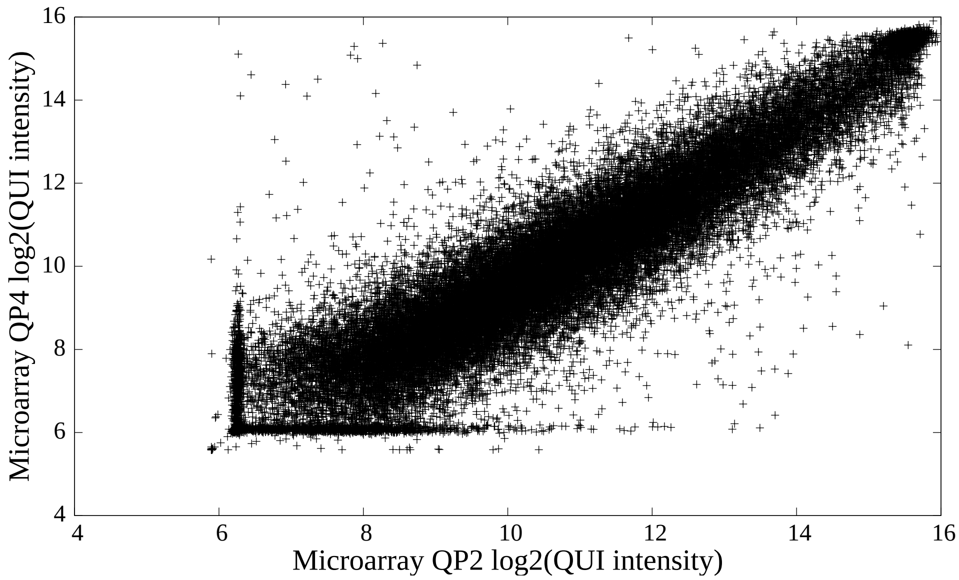

Supplementary Figure S11: The log-transformed QUI intensity values from microarray QP2 and microarray QP4 had a strong correlation ($r = 0.92$).

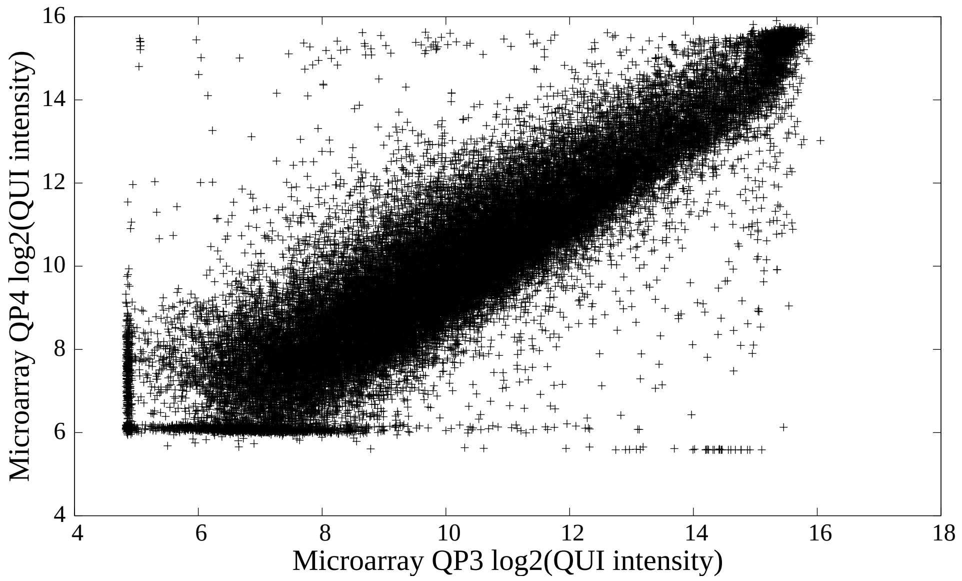

Supplementary Figure S12: The log-transformed QUI intensity values from microarray QP3 and microarray QP4 had a strong correlation ($r = 0.89$).

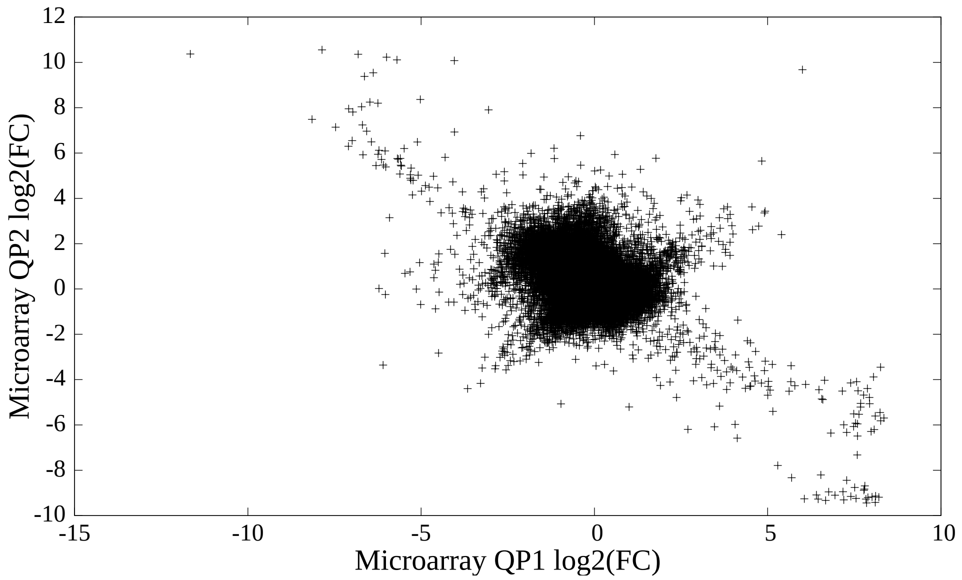

Supplementary Figure S13: The log-transformed FC values from microarray QP1 and microarray QP2 had a negative correlation ($r = -0.46$).

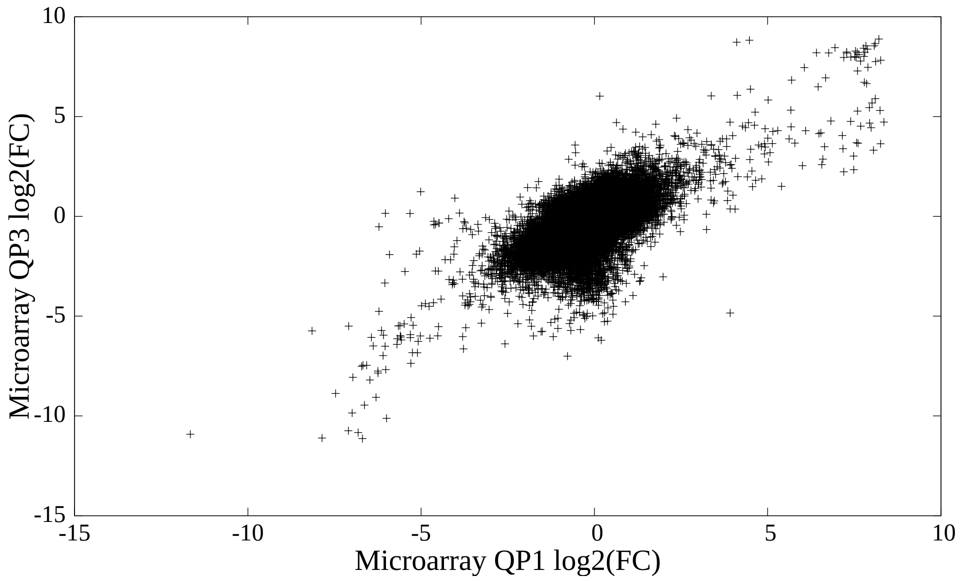

Supplementary Figure S14: The log-transformed FC values from microarray QP1 and microarray QP3 had a moderate to strong correlation ($r = 0.70$).

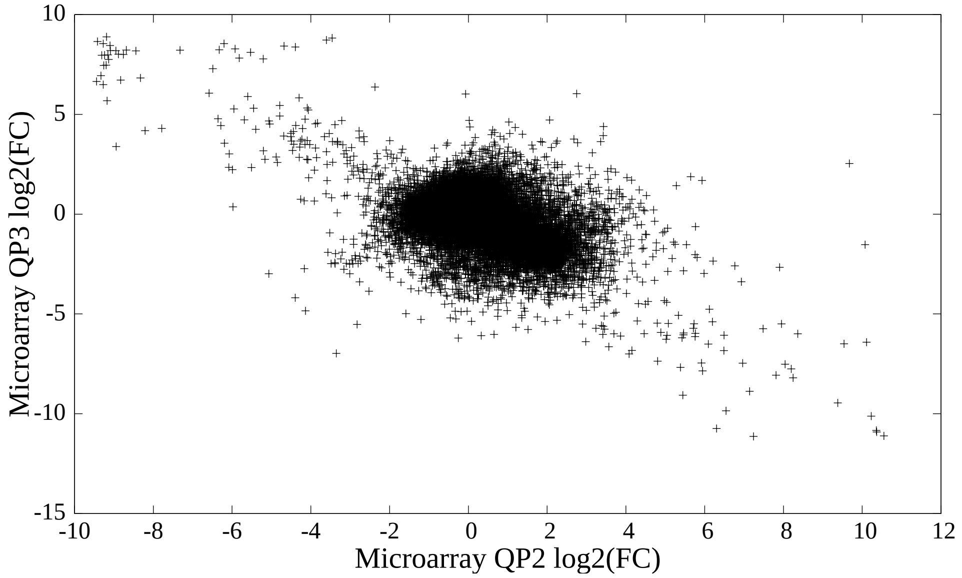

Supplementary Figure S15: The log-transformed FC values from microarray QP2 and microarray QP3 had a negative correlation ($r = -0.55$).

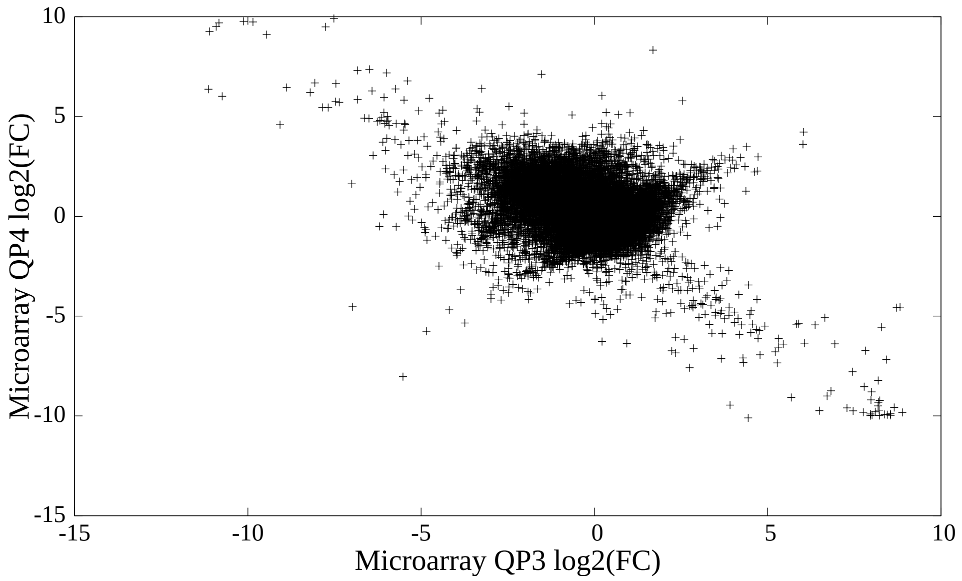

Supplementary Figure S16: The log-transformed FC values from microarray QP3 and microarray QP4 had a negative correlation ($r = -0.44$).

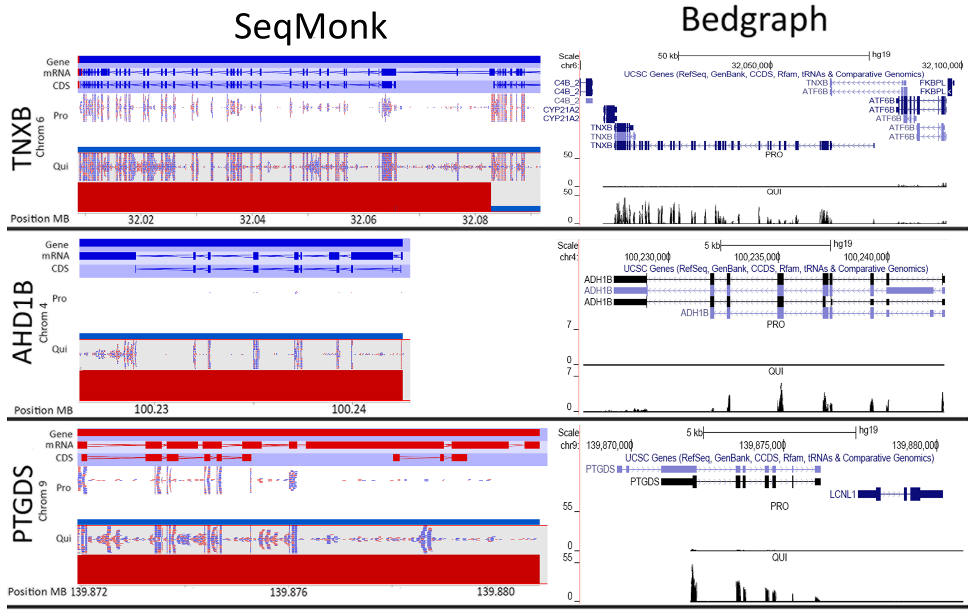

Supplementary Figure S17: RNA-seq reads from proliferative and quiescent samples. Mapped RNA-seq data were imported into SeqMonk and quantified using the RPKM method (left column). Reads for the *TNXB* (top panel), *AHD1B* (middle panel), and *PTGDS* (lower panel) genes are shown. For each gene, the gene length, mRNA (exons are shown as blocks with introns shown as arrowheads) and coding regions are indicated. Arrows and color indicate the direction of the gene on the chromosome (red, left to right; blue, right to left). Reads from proliferative (Pro) and quiescent (Qui) samples are shown (blue dash, right to left mapped read; red dash, left to right mapped read). Bars under the reads represent fold change; blue bar for proliferative reads is set to 1 as the baseline. Red bar under Qui reads represents the fold change

as compared to Pro reads; *TNXB* (Chromosome 6), 47-fold increase; *AHD1B* (Chromosome 4), 49-fold increase; *PTGDS* (Chromosome 9), 27-fold increase. Chromosome position is listed at the bottom of each panel. Mapped RNA-seq reads were imported into SeqMonk and the total numbers of reads at each base pair across the coding region of the gene quantified (right column). Quantification was imported as custom tracks into the UCSC genome browser and bedgraphs created for the *TNXB* (top panel), *AHD1B* (middle panel), and *PTDGS* (lower panel) genes. In each graph, the genomic location and scale are shown along with coding sequences (including intron and exons) for each UCSC gene. PRO and QUI indicate the bar graphs for the total reads for each sample. The scale for the number of reads is demonstrated on the left of the graph. Arrowheads indicate the direction of the genes on the chromosome. For the *TNXB* graph, the sequence from the *ATF6B* gene is shown to demonstrate near equivalent reads between the two samples. Arrowheads indicate the direction of the gene on the chromosome.

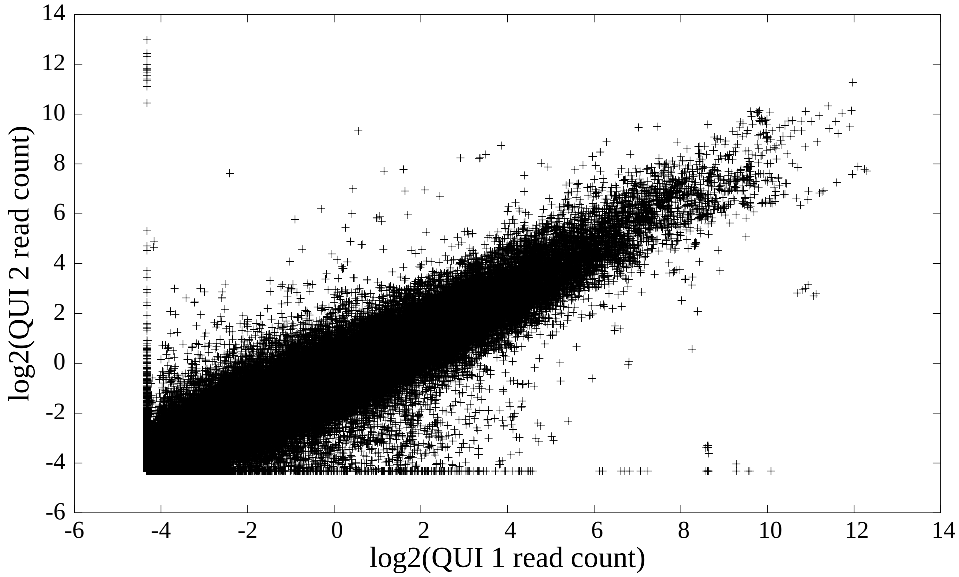

Supplementary Figure S18: The log-transformed read counts from the two RNA-seq reactions for the quiescent fibroblasts exhibited a strong linear relationship ($r = 0.94$).

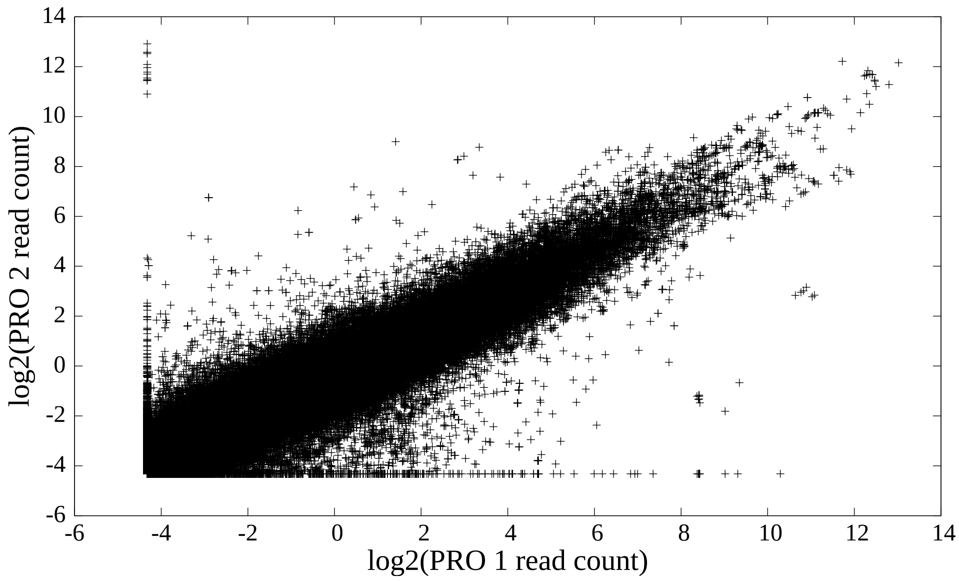

Supplementary Figure S19: The log-transformed read counts from the two RNA-seq reactions for the proliferative fibroblasts exhibited a strong linear relationship ($r = 0.94$).

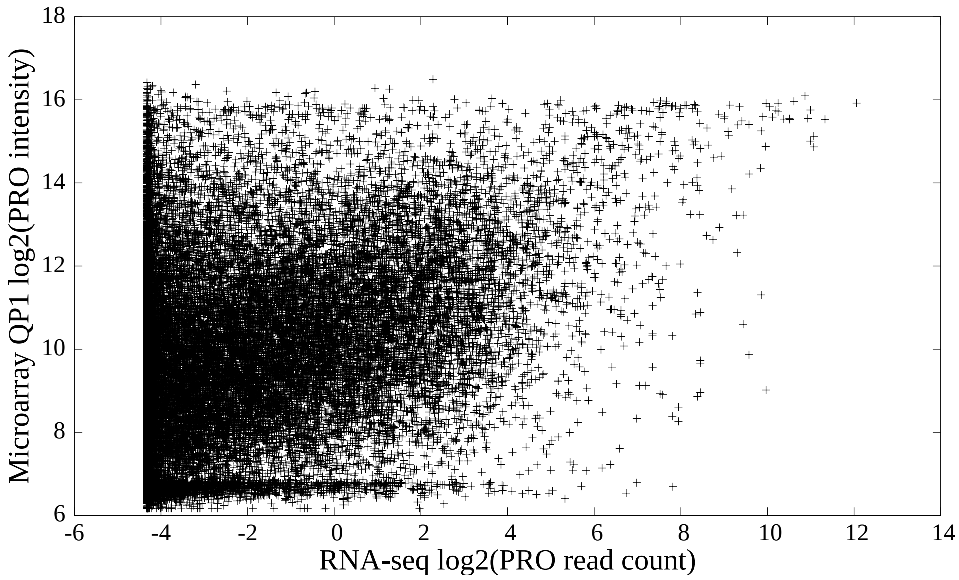

Supplementary Figure S20: The log-transformed RNA-seq read counts for PRO and the log-transformed PRO intensity values from microarray QP1 had a low correlation ($r = 0.33$).

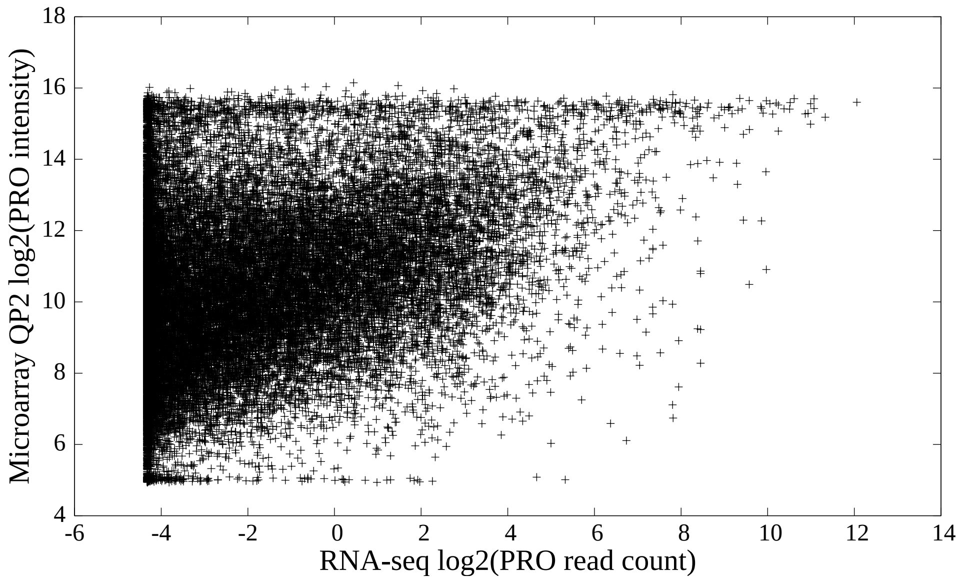

Supplementary Figure S21: The log-transformed RNA-seq read counts for PRO and the log-transformed PRO intensity values from microarray QP2 had a low correlation ($r = 0.33$).

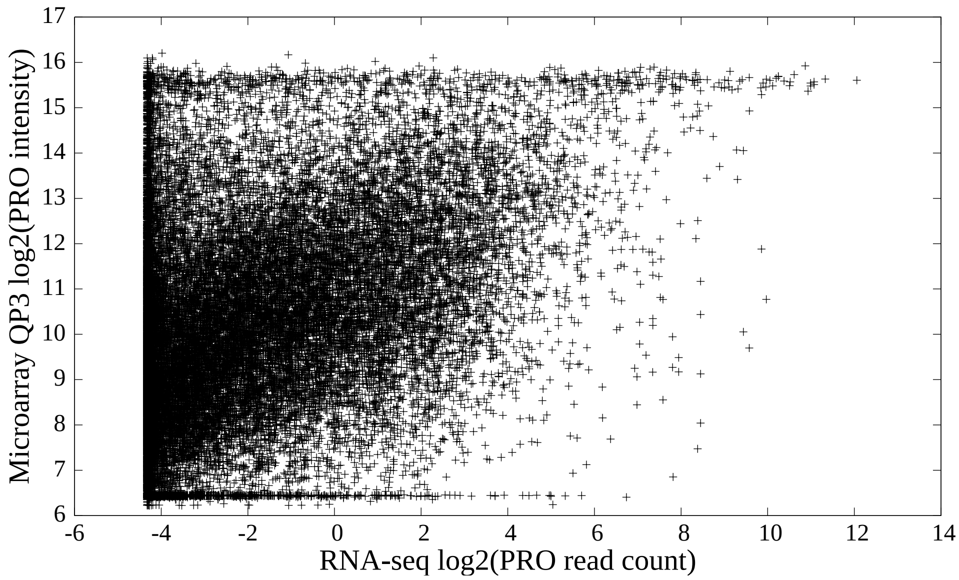

Supplementary Figure S22: The log-transformed RNA-seq read counts for PRO and the log-transformed PRO intensity values from microarray QP3 had a moderate correlation ($r = 0.40$).

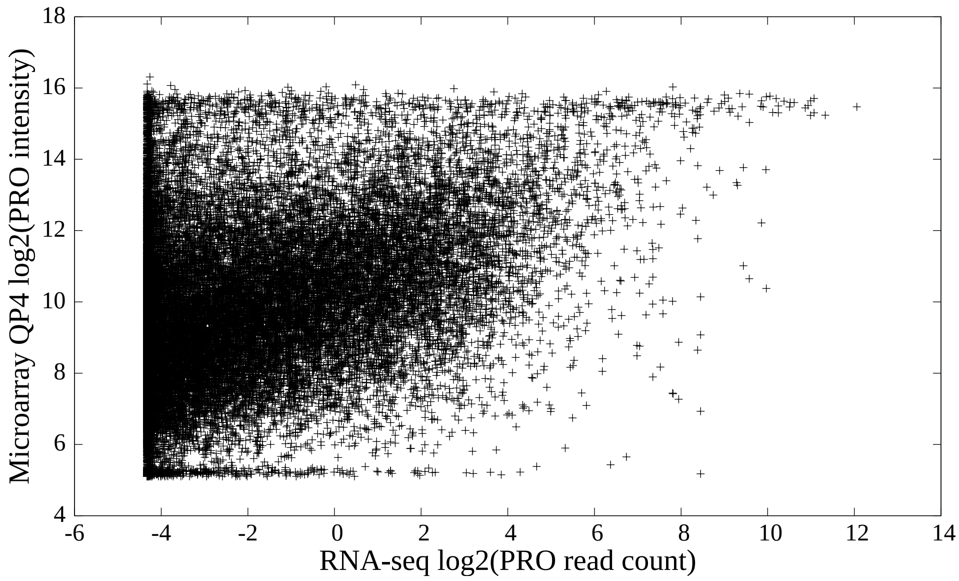

Supplementary Figure S23: The log-transformed RNA-seq read counts for PRO and the log-transformed PRO intensity values from microarray QP4 had a low correlation ($r = 0.37$).

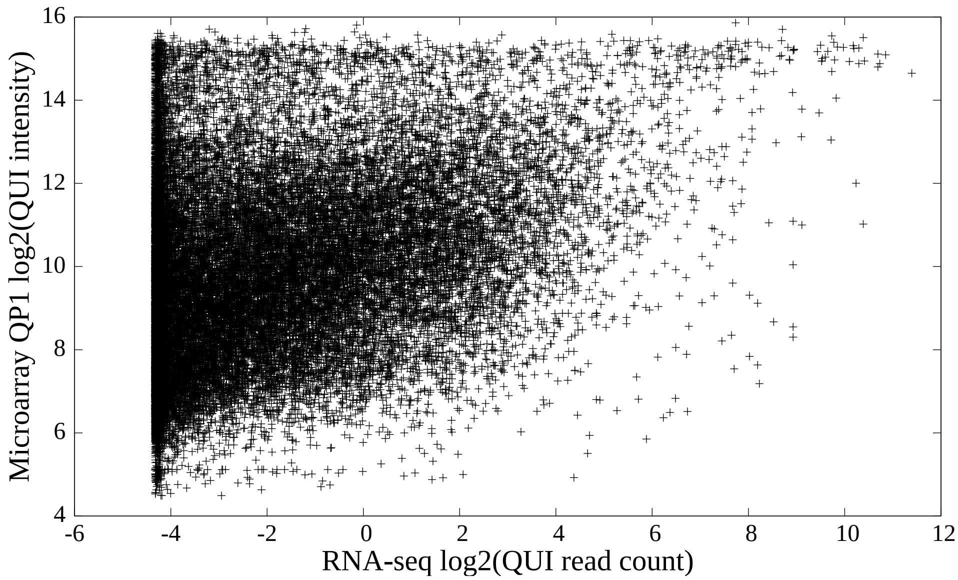

Supplementary Figure S24: The log-transformed RNA-seq read counts for QUI and the log-transformed QUI intensity values from microarray QP1 had a low correlation ($r = 0.32$).

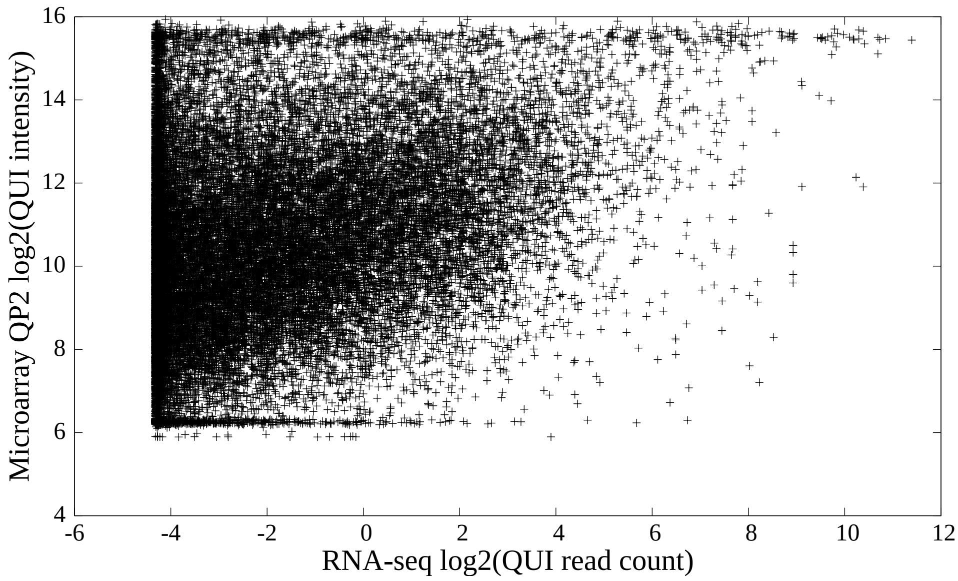

Supplementary Figure S25: The log-transformed RNA-seq read counts for QUI and the log-transformed QUI intensity values from microarray QP2 had a low correlation ($r = 0.32$).

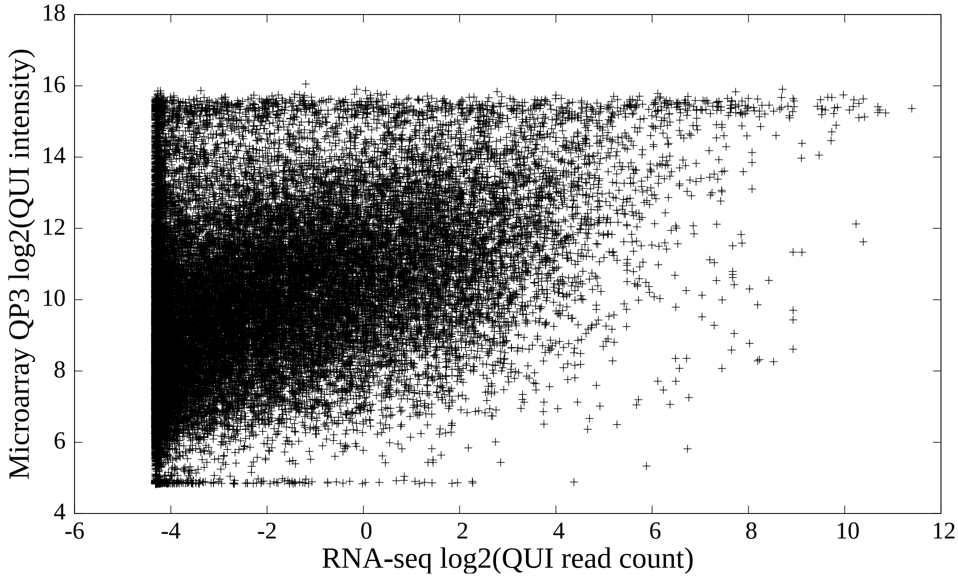

Supplementary Figure S26: The log-transformed RNA-seq read counts for QUI and the log-transformed QUI intensity values from microarray QP3 had a moderate correlation ($r = 0.41$).

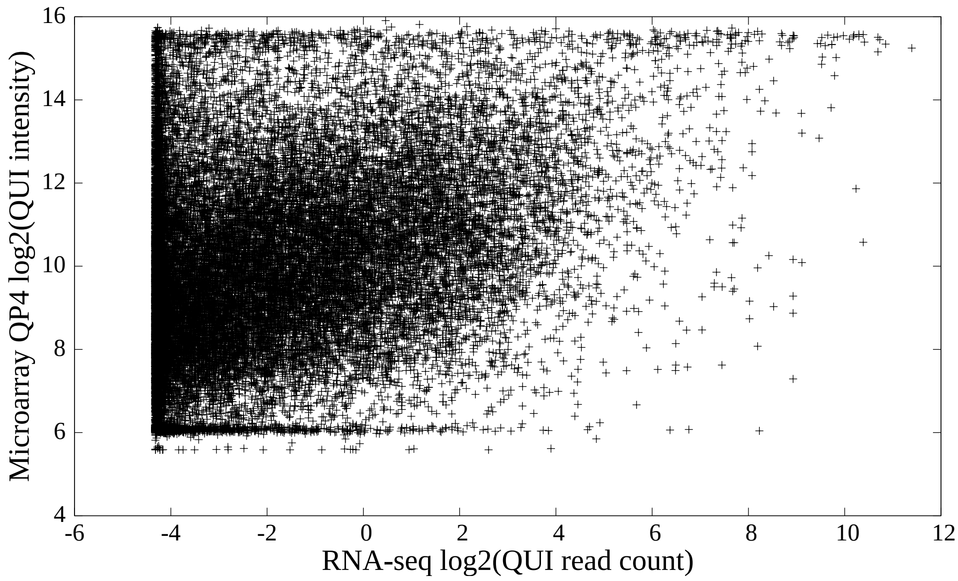

Supplementary Figure S27: The log-transformed RNA-seq read counts for QUI and the log-transformed QUI intensity values from microarray QP4 had a low correlation ($r = 0.35$).

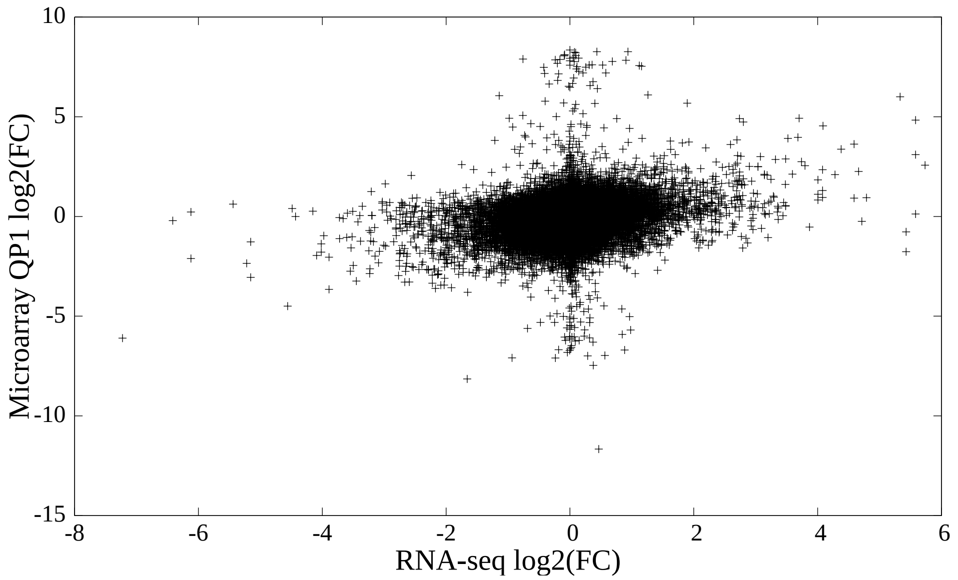

Supplementary Figure S28: The log-transformed RNA-seq FC values and the log-transformed FC values from microarray QP1 had a low correlation ($r = 0.23$).

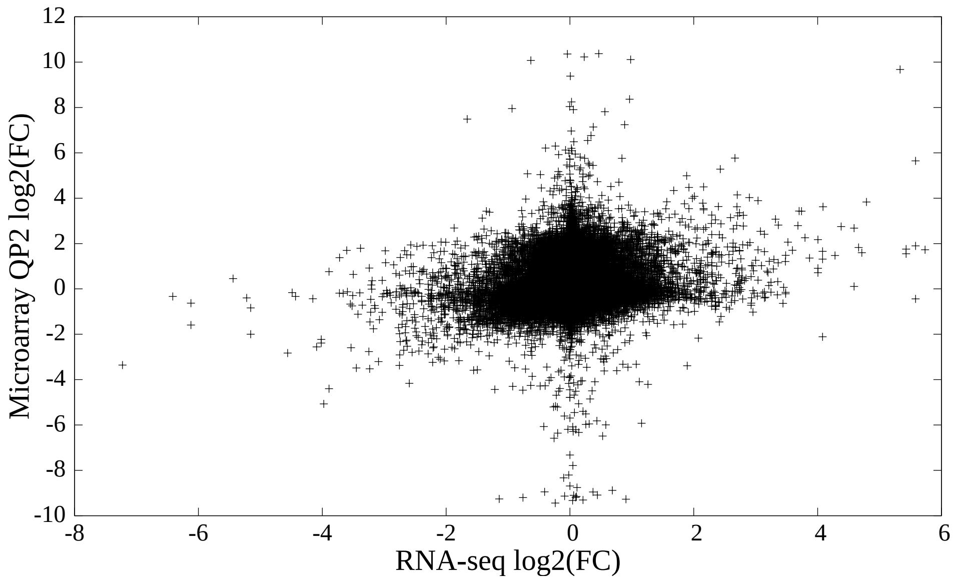

Supplementary Figure S29: The log-transformed RNA-seq FC values and the log-transformed FC values from microarray QP2 had a low correlation ($r = 0.18$).

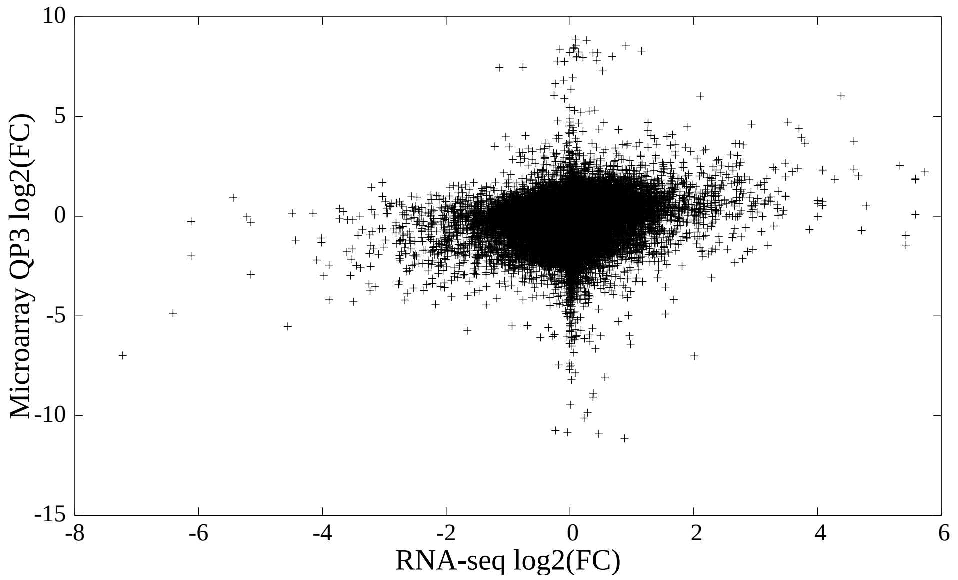

Supplementary Figure S30: The log-transformed RNA-seq FC values and the log-transformed FC values from microarray QP3 had a low correlation ($r = 0.18$).

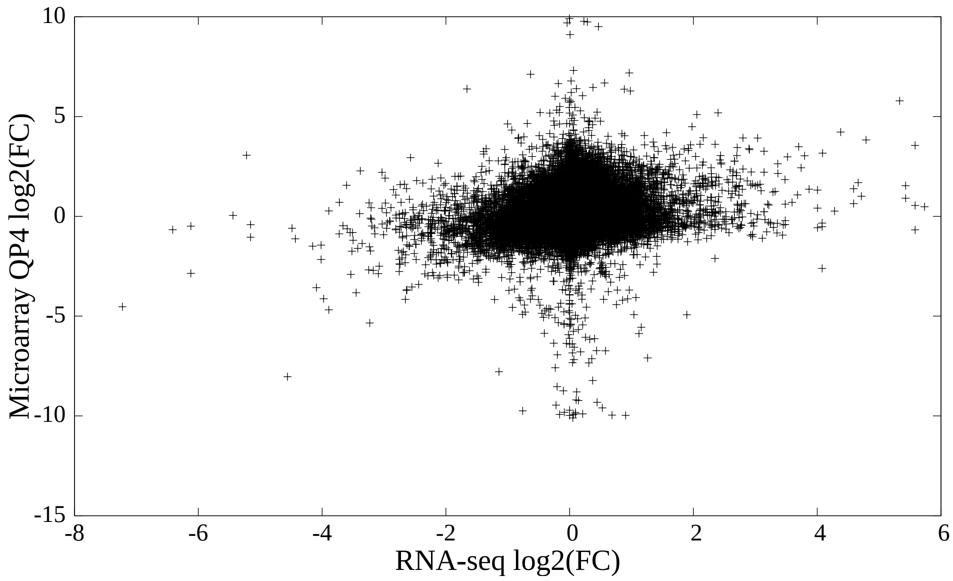

Supplementary Figure S31: The log-transformed RNA-seq FC values and the log-transformed FC values from microarray QP4 had a low correlation ($r = 0.17$).

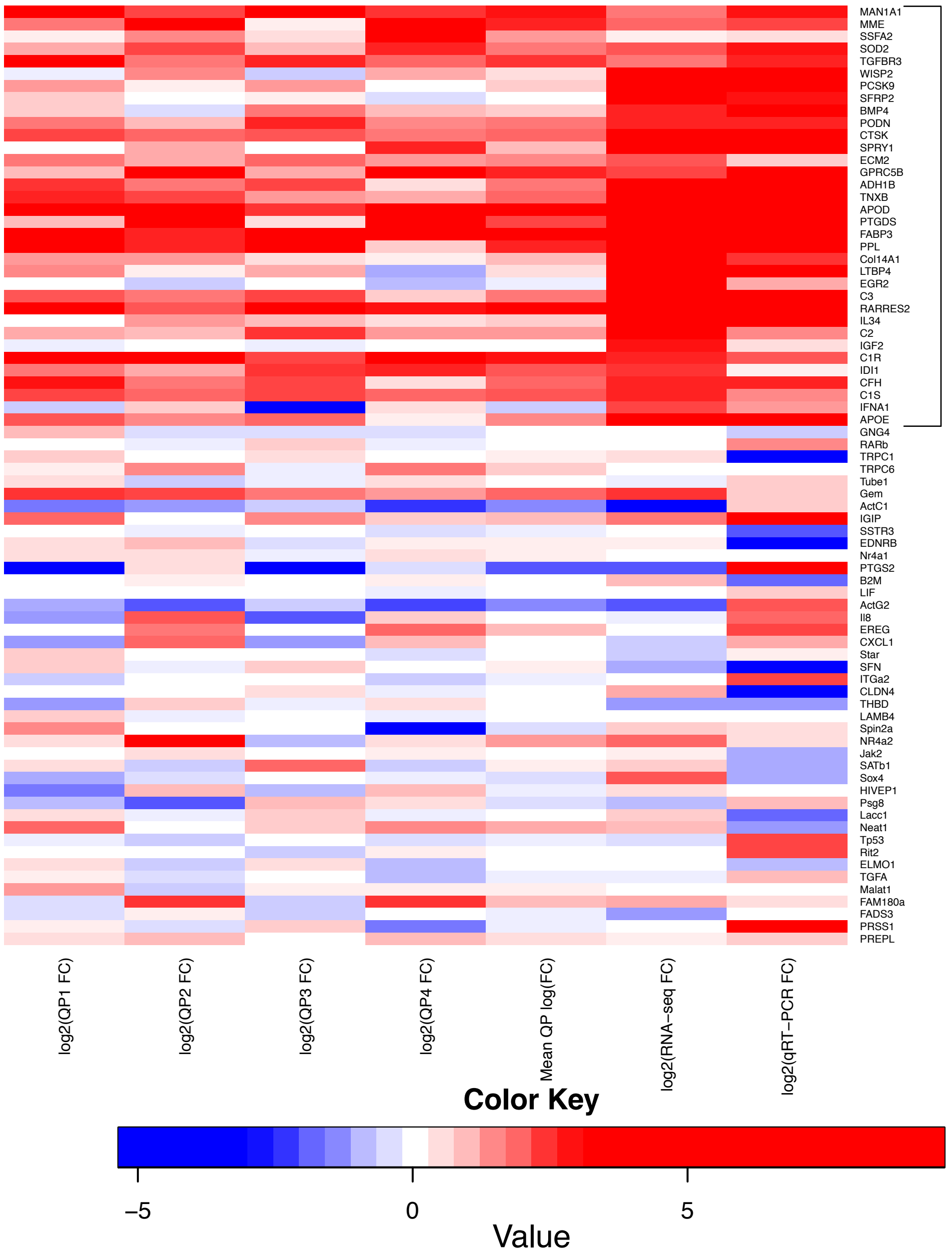

Supplementary Figure S32: Correspondence between the microarray data, the RNA-seq data, and the qRT-PCR data for the probes that were analyzed using qRT-PCR. Rows correspond to genes, while columns correspond to the source of the data (RNA-seq, qRT-PCR, one of the four microarrays, or the average of the four microarrays, which is denoted "Mean QP log2(FC)"). The color of each cell denotes the FC value for the corresponding gene and source. The bracketed genes were selected because they had a high FC value (QUI/PRO) in either the RNA-seq data, the microarray data, or both. Non-bracketed genes were selected arbitrarily. The raw values used to create this figure are given in Supplementary Table S6.

Supplementary Table S1: Microarray intensity values demonstrate high reproducibility. Correlations are shown for proliferative samples (PRO) or quiescent samples (QUI) for each pair of microarrays. For each cell state (PRO or QUI), the values in the upper diagonal contain the Pearson correlations, while those in the lower diagonal contain the Spearman correlations. Values not in parentheses represent correlations between intensity values after cross-channel correction and LOWESS normalization, while those in parentheses represent correlations after those values had also been log-transformed. As log transformation does not change the rank order, only one number is shown for the Spearman correlation for each pair. All correlations were ≥ 0.77, indicating strong agreement between intensity values in all microarrays.

	Signal intensities for PRO				Signal intensities for QUI			
	QP1	QP2	QP3	QP4	QP1	QP2	QP3	QP4
QP1	–	0.80 (0.89)	0.91 (0.89)	0.80 (0.87)	–	0.88 (0.91)	0.92 (0.90)	0.88 (0.89)
QP2	0.89	–	0.78 (0.78)	0.94 (0.94)	0.91	–	0.85 (0.85)	0.93 (0.92)
QP3	0.87	0.77	–	0.79 (0.80)	0.89	0.84	–	0.89 (0.89)
QP4	0.88	0.94	0.79	–	0.89	0.92	0.89	–

Supplementary Table S2: List of probes in common among the n microarray probes with the highest FC values (geometric mean; QUI/PRO) and the n RNA-seq probes with the highest FC values, where n = 10, 50, 100, 500, or 1000.

Microarray probe ID	RNA-seq probe ID
$n = 10$	
H200006183	APOD-001
H300018610	TNXB-001
$n = 50$	
H200006183	APOD-001
opHsV0400000146	FABP3-001
H300009988	LRRN4CL-001
H300018610	TNXB-001
H200004405	RARRES2-001
H200006911	GPNMB-001
H200014099	CFD-201
H300005569	PRELP-001
H200005933	PPL-001
opHsV0400003635	SBSN-001
H200006005	CLU-001
H300014703	FBLN1-001
$n = 100$	
H200006183	APOD-001
opHsV0400000146	FABP3-001
H300009988	LRRN4CL-001
H300018610	TNXB-001
H200004405	RARRES2-001
H200006911	GPNMB-001
H300004102	C1R-201
H200014099	CFD-201
opHsV0400006073	CFH-001
H300010096	AKR1C2-001
opHsV0400008625	C5orf4-001
H300004073	AKR1C1-001
H300005569	PRELP-001
H300018014	DCN-001
H200005933	PPL-001
opHsV0400003635	SBSN-001
opHsV0400004502	C1R-201
H200000063	FMOD-001
H200006005	CLU-001
H300014703	FBLN1-001
H200001631	PTGDS-001
H200016303	AC132217.4-001
H200009651	MFAP4-001
$n = 500$	
H200006183	APOD-001
opHsV0400000146	FABP3-001
H300009988	LRRN4CL-001
H300018610	TNXB-001

H200004405	RARRES2-001
H200006911	GPNMB-001
H300004102	C1R-201
H200014099	CFD-201
H300021913	AKR1C3-001
opHsV0400006073	CFH-001
H300010096	AKR1C2-001
opHsV0400008625	C5orf4-001
H200003498	MAN1A1-001
H300004073	AKR1C1-001
H300005569	PRELP-001
opHsV0400012696	HMGCS1-001
H300018014	DCN-001
H200008754	SLC39A8-001
H300001612	PCOLCE2-001
H200015788	GPRC5B-001
H200005933	PPL-001
opHsV0400005905	ADH1A-001
H200000338	MME-001
H200008154	SERPINF1-001
opHsV0400003635	SBSN-001
opHsV0400004502	C1R-201
H300009266	TGFBR3-001
H300012822	SOD2-002
opHsV0400006810	INSIG1-001
H200013052	FADS1-001
H200014059	MMP11-001
H200005322	ABI3BP-001
H200000063	FMOD-001
H200004290	ECM2-001
H200006005	CLU-001
opHsV0400004530	SCPEP1-201
H200000614	SOD3-001
H300014703	FBLN1-001
H200008006	SEMA3C-001
H200002453	SRPX-003
H200001631	PTGDS-001
opHsV0400004408	WLS-003
H300016592	FMO1-001
H200006162	GSTM5-001
H200016303	AC132217.4-001
opHsV0400001570	CDR1-001
H200001714	SVEP1-003
H300021699	ADH1A-001
H300010817	FAP-001
H200009651	MFAP4-001
H200017417	MGP-004
H200013978	SVIL-001
H300009117	OSR2-002
H200006954	RARRES1-001
H300019395	CD36-006

opHsV0400008225	SCD-001
H200005314	ADAMTS5-001
opHsV0400001713	C1S-003
opHsV0400001712	C1S-003
H200013753	SERPING1-001
H200000111	FAP-001
opHsV0400000317	IDI1-004
H300018881	HMGCS1-001
H200006607	GEM-001
H200007076	CTSK-001
opHsV0400005446	CFH-001
H200002762	HSD17B14-201
H300011049	PTGES-001
H200011160	ISLR-001
opHsV0400005369	TNXB-001
H200008602	PLSCR4-001
H300018456	DCN-001
H200019469	ORAI3-001
H300001186	DMKN-001
H300012124	FBLN1-001
H200012110	AEBP1-001
H200006485	CPZ-001
H300006936	TXNIP-001
opHsV0400012347	ADAMTS5-001
H300018640	APOL1-003
opHsV0400000736	GAA-201
H200006052	CTSO-001
opHsV0400000778	ADH1B-004
H300022455	LTBP3-001
H200019338	ANG-001
opHsV0400004681	PCSK9-001
H200005781	MXRA5-001
H200012213	NPC2-008
H200019931	CTSD-001
H300008317	SMPDL3A-001
opHsV0400000678	TPP1-001
H300004858	PODN-001
H200008806	C3-001
H200011421	CDKN1C-002
H200020242	IL33-001
H200014658	DIRAS3-001
H200007894	PDGFRL-001
H300015570	FBLN2-002
H200006728	LUM-001
H300016767	PSAP-002
opHsV0400004093	ECM2-001
H200014829	FBLN2-002
H300019693	SVEP1-002
opHsV0400012949	LYNX1-001
opHsV0400000297	ASAH1-001
H200006022	CHI3L1-001

opHsV0400003457	SERPING1-001
opHsV0400005637	DPP4-001
H300014091	COL14A1-001
H300021118	BBC3-201
H200018923	SOD2-002
H200011779	FCGRT-201
opHsV0400010879	AKAP12-202
H200005403	HTRA3-001
H200007822	APOE-001
H200006553	VEGFB-001
H300016877	C2-001
H300019463	RALGPS1-001
H200007999	GBP2-002
H200000817	AGT-001
H300010511	SOD2-002
opHsV0400005864	SEPP1-001
H200013247	VIT-001
H200003669	PIK3IP1-001
H200006891	NR4A2-001
H200009720	GDF15-201
H200007307	GPR133-001
H300005309	TMEM35-001
H200008267	CPXM1-001
H300018457	GRN-201
H200011392	SFRP4-001
$n = 1000$	
H200006183	APOD-001
opHsV0400000146	FABP3-001
H300009988	LRRN4CL-001
H300018610	TNXB-001
H200004405	RARRES2-001
H200006911	GPNMB-001
H300004102	C1R-201
H200014099	CFD-201
H300021913	AKR1C3-001
opHsV0400006073	CFH-001
H300010096	AKR1C2-001
opHsV0400008625	C5orf4-001
H200003498	MAN1A1-001
H300004073	AKR1C1-001
H300005569	PRELP-001
opHsV0400012696	HMGCS1-001
H300018014	DCN-001
H200008754	SLC39A8-001
H300001612	PCOLCE2-001
H200015788	GPRC5B-001
H200005933	PPL-001
opHsV0400005905	ADH1A-001
H200000338	MME-001
H200008154	SERPINF1-001
opHsV0400003635	SBSN-001

opHsV0400004502	C1R-201
H300009266	TGFBR3-001
H200003092	FAM198B-001
H300012822	SOD2-002
H300002477	RCAN2-001
opHsV0400006810	INSIG1-001
H200001408	SFRP1-001
H200013052	FADS1-001
H200014059	MMP11-001
H200005322	ABI3BP-001
H200000063	FMOD-001
H200004290	ECM2-001
H200006005	CLU-001
opHsV0400004530	SCPEP1-201
H200000614	SOD3-001
H300014703	FBLN1-001
H200008006	SEMA3C-001
H200002453	SRPX-003
H200001631	PTGDS-001
opHsV0400004408	WLS-003
H300016592	FMO1-001
H200006162	GSTM5-001
H200016303	AC132217.4-001
opHsV0400001570	CDR1-001
H200001714	SVEP1-003
H300021699	ADH1A-001
H300010817	FAP-001
H200009651	MFAP4-001
H200017417	MGP-004
H200013978	SVIL-001
H300009117	OSR2-002
H200006954	RARRES1-001
H300019395	CD36-006
opHsV0400008225	SCD-001
H200005314	ADAMTS5-001
opHsV0400001713	C1S-003
opHsV0400010717	SEMA5A-001
opHsV0400001712	C1S-003
H200013753	SERPING1-001
H200000111	FAP-001
opHsV0400000317	IDI1-004
H300018881	HMGCS1-001
H300015355	BOC-003
H200001091	CREG1-001
H200006607	GEM-001
H200007076	CTSK-001
opHsV0400005446	CFH-001
H200003843	SAT1-001
H200005947	GJA1-001
H200001130	SLC40A1-001
H200002762	HSD17B14-201

H300022366	CCDC74B-001
H300011049	PTGES-001
H200011160	ISLR-001
opHsV0400005369	TNXB-001
H200008602	PLSCR4-001
H300018456	DCN-001
H200005026	HEXB-001
H200019469	ORAI3-001
H300001186	DMKN-001
H300012124	FBLN1-001
H200012110	AEBP1-001
H200006485	CPZ-001
H300006936	TXNIP-001
opHsV0400012347	ADAMTS5-001
H200009211	SC5DL-001
H200013953	BACH1-001
H300018640	APOL1-003
opHsV0400000736	GAA-201
opHsV0400001994	RCAN2-001
H200006052	CTSO-001
opHsV0400000778	ADH1B-004
H300022455	LTBP3-001
H200019338	ANG-001
opHsV0400004681	PCSK9-001
H200005781	MXRA5-001
opHsV0400003760	SAT1-001
H200012213	NPC2-008
H200019931	CTSD-001
H300008317	SMPDL3A-001
opHsV0400000678	TPP1-001
H200019102	ADD3-001
H300004858	PODN-001
H200008806	C3-001
H200011421	CDKN1C-002
H200020242	IL33-001
H200014658	DIRAS3-001
H200007894	PDGFRL-001
H300015570	FBLN2-002
H200006728	LUM-001
H300016767	PSAP-002
opHsV0400012444	FBXO32-001
H200006008	HTRA1-001
opHsV0400004093	ECM2-001
H200014829	FBLN2-002
H300019693	SVEP1-002
H200006869	GYPC-001
H200004859	F10-001
H200016555	IFI6-001
opHsV0400012949	LYNX1-001
H200004464	MYO1D-201
H300015674	SIAE-001

H300022324	ADAMTS13-001
opHsV0400000297	ASAH1-001
H300018814	PMP22-002
H200006022	CHI3L1-001
opHsV0400003457	SERPING1-001
opHsV0400005637	DPP4-001
H300014091	COL14A1-001
H200018907	TRIM22-001
H300021118	BBC3-201
H200018923	SOD2-002
H300015796	LSS-002
H200010692	FAM8A1-001
H200011779	FCGRT-201
opHsV0400005894	ALDH3A2-001
opHsV0400010879	AKAP12-202
H200005403	HTRA3-001
H200007822	APOE-001
H200006553	VEGFB-001
H200012068	SLC17A5-001
H300019720	LGALS3BP-201
H200014286	DRAM1-001
H300016877	C2-001
H200015797	RHOJ-001
H300019463	RALGPS1-001
H200017054	IGFBP6-001
H200014757	PGCP-001
H200015671	ITM2B-001
H200007999	GBP2-002
H200016211	ZNF219-001
H200000817	AGT-001
opHsV0400002899	THBS2-001
H300010511	SOD2-002
opHsV0400005864	SEPP1-001
H200013247	VIT-001
opHsV0400004072	MYO1D-201
H200006628	CCNG1-001
opHsV0400000112	CCNG1-001
H200003669	PIK3IP1-001
H200006891	NR4A2-001
H300022033	PDZRN3-001
H200005977	PRNP-002
opHsV0400005051	COL6A2-001
H300000866	LRRC32-001
H200009720	GDF15-201
H300019371	DDB2-201
H300011696	TGFBR2-001
H200007307	GPR133-001
H300005309	TMEM35-001
opHsV0400006111	HIST1H2AC-001
H200008267	CPXM1-001
H300018457	GRN-201

H200011392	SFRP4-001
opHsV0400010785	SLC40A1-001
opHsV0400000770	CST1-001
H300007158	CLDN11-001
H200001525	C7orf41-001
H200001162	ARHGAP20-001
H200015691	MSMO1-001
H300011496	FBLN2-002
opHsV0400012025	AC159540.1-001
H300018970	SESN1-002
opHsV0400007437	RP11-152F13.5-201
H200001709	QPRT-201
H200005879	BMP2-001
H200017694	PCDHB6-001
H200007939	PCSK6-203
H300006979	NBL1-001
opHsV0400004676	SLIT3-002
opHsV0400006369	C14orf132-001
H200016323	ZMAT3-001
opHsV0400000698	COL6A2-001
H200013144	CST3-001
H300002218	LRIG3-001
H300019462	KLF4-002
H300017367	CFHR1-001
H200011747	MMP2-001
H300007781	RARRES3-001
H300022100	ANK1-001
opHsV0400011450	ZMAT3-001
opHsV0400003010	PLD3-004
H300018477	PTPRU-001
H200006117	TP53INP1-001
H300017119	FAM46A-003
H300011586	PMP22-002
opHsV0400004882	TCEAL7-001
H200010452	C17orf58-201
H200002015	CTSF-001
H300003822	AC135178.1-201
H200007928	TFPI-201
H300021603	FKBP9-001
H200008758	TSHZ1-001
H300000904	AKAP12-001
H200003862	HIST1H2AC-201
H200007344	RENBP-004
H300010195	CCDC74A-001
H300006654	ZNFX1-AS1-001
opHsV0400012080	MBP-001
H300018991	PGF-002
opHsV0400000735	GAA-201
H300009961	SPATA18-001
H200020465	FAM102A-001
H200006772	DPT-001

H200004653	SULF2-001
H200004209	RAMP1-001
H200005091	CD81-001
H300011592	SH3D19-201
opHsV0400002187	KRBA2-001
H200009585	IGIP-002
H300003004	PDPN-001
H300019123	GLTSCR2-201
H200017774	PCDHB14-001
H200002317	RAB3IL1-002
H200004335	C19orf76-201
H300018539	CTSA-001
opHsV0400003366	IFITM10-001
H200006892	LAMB1-001
opHsV0400004289	APP-001
H300007451	FIBIN-001
H200006910	CHRDL1-001
H200008162	ADAM33-001
opHsV0400006844	SPRY1-002
opHsV0400003609	ADAM33-001
opHsV0400004448	PNRC1-001
H200005320	PLEKHF1-201
H200000520	TGFB3-001
H300013304	RNASET2-007
H200017630	PLTP-001
H200007742	BTN3A3-001
H300006701	C13orf16-002
H200000114	FLT3LG-201

Supplementary Table S3: Moderate overlap between the probes with the lowest FC values in the RNA-seq data and those with the lowest FC values in the DNA microarray data. k represents the size of a given list (the 10, 50, 100, 500, or 1000 probes with the lowest FC values), while n represents the number of probes in common between a list from the RNA-seq data and the corresponding list from the DNA microarray. The P-value represents the proportion of 10,000 random trials that had an equal or greater level of overlap than that actually observed. Thus, if none of the random trials had a greater level of overlap, then the P-value is 0. More overlapping probes than would be expected by chance were observed for all microarrays for $k = 100$, 500, and 1000. "All" represents the geometric mean of the FC values of the four microarrays; for this dataset, more overlapping probes were detected than would be expected by chance for all list sizes.

	$k = 10$		$k = 50$		$k = 100$		$k = 500$		$k = 1000$	
	n	P-value	n	P-value	n	P-value	n	P-value	n	P-value
QP1	0	1	1	0.08	6	0	74	0	156	0
QP2	0	1	1	0.08	8	0	91	0	214	0
QP3	0	1	2	0.002	5	0	41	0	92	0
QP4	0	1	1	0.08	6	0	78	0	183	0
All	2	0	13	0	31	0	156	0	316	0

Supplementary Table S4: List of probes in common among the n microarray probes with the lowest FC values (geometric mean; QUI/PRO) and the n RNA-seq probes with the lowest FC values, where $n = 10$, 50, 100, 500, or 1000.

Microarray probe ID	RNA-seq probe ID
$n = 10$	
H200019509	BEX1-001
H200006097	ID1-001
$n = 50$	
H200019509	BEX1-001
H200006097	ID1-001
H200007186	FAM64A-201
H200007019	MMP3-001
H200009826	KRT34-201
H200013276	SERPINB7-201
H200015043	CENPM-001
H200006195	TAGLN-006
H300006172	CDC20-001
H200006884	SERPINE1-001
opHsV0400006470	KRTAP1-1-001
H200018730	KRTAP1-5-001
H200018729	KRTAP2-3-001
$n = 100$	
H200019509	BEX1-001
H200006097	ID1-001
H200007186	FAM64A-201
H200007019	MMP3-001
H200009826	KRT34-201
H200013276	SERPINB7-201
H300019126	WNK4-201
opHsV0400000209	GINS2-001
H200000064	FOXM1-001
opHsV0400001096	TPM1-001

H200015043	CENPM-001
H300006703	LY6K-001
H200006195	TAGLN-006
H300006172	CDC20-001
H300018013	LOXL2-001
H200006884	SERPINE1-001
opHsV0400006470	KRTAP1-1-001
H200018730	KRTAP1-5-001
H200014659	CCNB2-001
H300018499	TRIP13-001
H200018729	KRTAP2-3-001
H300022133	TPM1-005
H300019521	MYPN-001
H200006072	RRM2-001
H200003347	CCNB1-001
H300018850	CDCA3-006
H200004906	CDCA8-001
H200010620	IL6-201
H200005562	KRT18-002
H200005476	F3-001
opHsV0400002741	F3-001
$n = 500$	
H200019509	BEX1-001
H200006097	ID1-001
H200007186	FAM64A-201
H200007019	MMP3-001
H200009826	KRT34-201
H200013276	SERPINB7-201
H300019126	WNK4-201
opHsV0400000209	GINS2-001
H300020373	TRIM55-001
opHsV0400005775	PRC1-003
H300008454	PTTG1-002
opHsV0400000040	MCM5-001
H200000064	FOXM1-001
H200015606	IL7R-001
opHsV0400001096	TPM1-001
H200015043	CENPM-001
H300006703	LY6K-001
H200006195	TAGLN-006
H300006172	CDC20-001
H200019588	CDK1-008
opHsV0400010150	C5orf13-001
H200010905	BUB1-001
H300006447	MT1G-001
H200002395	CEP55-001
H300018013	LOXL2-001
H300001203	GLIPR1-001
H300009655	MT1A-201
H200006884	SERPINE1-001
opHsV0400004690	HN1-003

H200006861	KIAA0101-001
opHsV0400006470	KRTAP1-1-001
H200018730	KRTAP1-5-001
H300013149	CDKN3-001
H200014659	CCNB2-001
H300018499	TRIP13-001
H300008477	FABP5-001
H200016257	STMN1-001
H200018729	KRTAP2-3-001
H300022133	TPM1-005
H300007298	C7orf74-001
H200014737	PTGS2-001
H300021553	NQO1-001
H300019609	NQO1-001
H300008824	MT1X-001
H300019521	MYPN-001
H200014949	HMOX1-001
opHsV0400005772	IQGAP3-001
H200012382	GTSE1-201
H200006072	RRM2-001
H200012451	SHCBP1-001
H300020154	RP11-566K11.2-202
H300007657	CKS2-001
H200004941	LPXN-001
opHsV0400001663	ARSI-001
H200003347	CCNB1-001
H300018850	CDCA3-006
H200004906	CDCA8-001
H200010620	IL6-201
H200005562	KRT18-002
H200008595	ACTN4-001
H200005476	F3-001
H200006619	MAD2L1-001
H200004336	WFDC1-001
H300012606	TRIM55-001
H300006471	MT1B-001
opHsV0400002741	F3-001
H200012135	NME1-002
H200016309	CTPS-001
opHsV0400005559	FAM83D-001
H300003230	EIF5A-001
H300019718	BIRC5-201
opHsV0400000170	MT1X-001
H200005926	MT1E-001
H200011714	SOCS2-001
H200006478	ACTG2-001
H200006177	SERPINB2-001
H200008131	ELL2-001
H200007139	CCNA2-001
H200009494	TNC-001
opHsV0400011316	CCDC99-001

opHsV0400000552	ACTC1-001
H200014300	SLC7A5-001
opHsV0400000924	UBE2S-201
opHsV0400006176	MALL-001
H300003254	TM4SF1-001
H200011549	CSRP1-001
H300003913	TOMM34-001
opHsV0400000171	VASP-201
H200006032	ODC1-001
H300006252	KRTAP2-2-001
H300005118	PKMYT1-001
H200007088	CDKN3-001
H300001422	CDT1-201
H300005177	EBNA1BP2-001
H300016261	CDK2-001
opHsV0400001053	EIF5AL1-001
H300009765	SKA1-001
H300008566	MT1M-201
opHsV0400001284	KRT16-001
H200003504	RNASEH2A-201
H200007136	WDR1-001
H300019778	PFKP-003
H200006415	PSRC1-001
H200014980	RHOB-001
opHsV0400008974	RAN-201
H200004769	HIST1H4C-001
H200010125	RACGAP1-001
H300013069	ANLN-001
H200006537	GGH-001
H300005337	DRAP1-001
opHsV0400004474	EIF5A-001
H200006882	CITED2-001
H200001767	TPX2-001
H200009647	TFPI2-001
H300013966	PPP1R14B-001
H200002233	ORMDL2-001
H200011329	PBK-001
opHsV0400006835	TGM2-002
H300013865	PSG5-001
H300020208	CYR61-201
opHsV0400000805	KISS1-001
H200003577	C5orf30-001
H300009991	RAB3B-001
H300006913	UBE2S-201
opHsV0400004298	MCM7-001
H200018834	DDX39A-201
H200019704	TUBA1B-001
opHsV0400000182	GCAT-001
H300019578	MAP2K3-001
H300012420	CENPA-001
H300021126	TUBA1C-001

H300003894	TOMM40-201
H300021305	MT1E-001
H200006466	TPM1-001
opHsV0400004073	MKI67-001
H200008018	NUAK2-001
H200004400	STRA13-001
H300021488	DTYMK-001
H300001995	HBEGF-001
H200012217	AP2S1-201
opHsV0400003358	TNC-001
opHsV0400003406	TUBB4B-001
opHsV0400013002	IL7R-001
opHsV0400000345	WDR1-013
opHsV0400000753	AC131180.1-201
H200002935	KIFC1-001
H200002802	CDK2-001
H300007069	SEMA7A-001
H200016720	KIF23-003
H200004166	CCDC99-001
H200005367	POC1A-001
H200002187	TIPARP-001
H200002810	CRIM1-001
H200011653	CORO1A-001
H200007833	ALDH1B1-001
opHsV0400002502	TUBA1B-002
$n = 1000$	
H200019509	BEX1-001
H200006097	ID1-001
H200007186	FAM64A-201
H200007019	MMP3-001
H200009826	KRT34-201
H200013276	SERPINB7-201
H300019126	WNK4-201
opHsV0400000209	GINS2-001
H300020373	TRIM55-001
opHsV0400005775	PRC1-003
H300008454	PTTG1-002
opHsV0400000040	MCM5-001
H300015276	CENPN-001
H200000064	FOXM1-001
H200015606	IL7R-001
opHsV0400001096	TPM1-001
opHsV0400004738	ANXA2-006
H200015043	CENPM-001
H300006703	LY6K-001
H200006195	TAGLN-006
H300006172	CDC20-001
H200019588	CDK1-008
opHsV0400010150	C5orf13-001
H200010905	BUB1-001
H300006447	MT1G-001

H200002395	CEP55-001
H300018013	LOXL2-001
H300001203	GLIPR1-001
H300009655	MT1A-201
H200017789	BEX2-002
H200006884	SERPINE1-001
opHsV0400004690	HN1-003
H200006861	KIAA0101-001
opHsV0400006470	KRTAP1-1-001
H200018730	KRTAP1-5-001
H200015506	NUF2-001
H300013149	CDKN3-001
H200014659	CCNB2-001
H300018499	TRIP13-001
H300008477	FABP5-001
H200016257	STMN1-001
H200018729	KRTAP2-3-001
H300022133	TPM1-005
H300007298	C7orf74-001
H200014737	PTGS2-001
H300021553	NQO1-001
H300019609	NQO1-001
H300008824	MT1X-001
H300019521	MYPN-001
H200014949	HMOX1-001
opHsV0400005772	IQGAP3-001
H200012382	GTSE1-201
H200006072	RRM2-001
H200006436	DLGAP5-001
H200012451	SHCBP1-001
H300020154	RP11-566K11.2-202
H300007657	CKS2-001
H200004941	LPXN-001
opHsV0400001663	ARSI-001
H200003347	CCNB1-001
H300018850	CDCA3-006
H200004906	CDCA8-001
H200010620	IL6-201
H200005562	KRT18-002
H200008595	ACTN4-001
H200005476	F3-001
H300022008	KIF4A-001
H200006619	MAD2L1-001
H300021996	DEPDC1-001
H200004336	WFDC1-001
H300012606	TRIM55-001
H300006471	MT1B-001
opHsV0400002741	F3-001
H300006162	RP11-480I12.5-002
H200012135	NME1-002
H200016309	CTPS-001

opHsV0400006230	SMTN-001
opHsV0400005559	FAM83D-001
H300003230	EIF5A-001
H300019718	BIRC5-201
opHsV0400000170	MT1X-001
H200005926	MT1E-001
H200011714	SOCS2-001
H200006478	ACTG2-001
H200006177	SERPINB2-001
opHsV0400005248	FAM46B-001
H200008131	ELL2-001
H200007139	CCNA2-001
H200009494	TNC-001
opHsV0400011316	CCDC99-001
opHsV0400000552	ACTC1-001
H200014300	SLC7A5-001
opHsV0400000924	UBE2S-201
opHsV0400006176	MALL-001
H300003254	TM4SF1-001
H200011549	CSRP1-001
H300003913	TOMM34-001
H200017742	LRRC59-001
opHsV0400000171	VASP-201
H200006032	ODC1-001
H300006252	KRTAP2-2-001
H300005118	PKMYT1-001
H200007088	CDKN3-001
H300001422	CDT1-201
H300012042	ITGA6-001
H300005177	EBNA1BP2-001
H300004589	ULBP2-001
H300016261	CDK2-001
H200006368	SLC22A4-001
opHsV0400001053	EIF5AL1-001
H300009765	SKA1-001
H300021066	CENPN-001
H300008566	MT1M-201
opHsV0400001284	KRT16-001
H200003504	RNASEH2A-201
H200007136	WDR1-001
H300019778	PFKP-003
H200006415	PSRC1-001
H200005149	UBASH3B-001
H200019789	TUBB2A-001
H200014980	RHOB-001
opHsV0400008974	RAN-201
H200004769	HIST1H4C-001
H200010125	RACGAP1-001
H300013069	ANLN-001
H200006537	GGH-001
H200000046	CDH2-001

H300005337	DRAP1-001
H200001306	PAFAH1B3-201
opHsV0400004474	EIF5A-001
H200006882	CITED2-001
H200001011	UBE2T-001
H300004091	UCK2-001
H200001767	TPX2-001
H200017739	CENPN-001
H200009647	TFPI2-001
H300013966	PPP1R14B-001
H200001623	ADAMTS1-001
H200008124	SNRPA-201
H200002233	ORMDL2-001
H200011329	PBK-001
opHsV0400006835	TGM2-002
opHsV0400000022	MOK-001
opHsV0400006275	NCAPD2-001
H300013865	PSG5-001
H300020208	CYR61-201
opHsV0400000805	KISS1-001
H200004554	MXD3-201
H200003577	C5orf30-001
H300009991	RAB3B-001
H300010998	TRIM14-001
H300006913	UBE2S-201
opHsV0400004298	MCM7-001
H200018834	DDX39A-201
H200019704	TUBA1B-001
H300020005	IMPDH1-001
opHsV0400000182	GCAT-001
H200007917	GOT2-001
opHsV0400006195	TUBA4A-001
H200003005	ZWILCH-001
H300019578	MAP2K3-001
H300004895	UBE2N-001
H300012420	CENPA-001
H300022630	DPP3-201
H300021126	TUBA1C-001
H200018176	CCL2-001
H300003894	TOMM40-201
H300021305	MT1E-001
H200006466	TPM1-001
opHsV0400004073	MKI67-001
H200008018	NUAK2-001
H200004400	STRA13-001
H300021488	DTYMK-001
opHsV0400001426	PPME1-001
H300001995	HBEGF-001
H200010574	C6orf125-001
H200012217	AP2S1-201
H200013972	MCM4-001

H200004950	ORC6-001
H300017348	ZYX-001
opHsV0400003358	TNC-001
H200010178	SRSF2-201
opHsV0400003406	TUBB4B-001
H300001508	RPL22L1-001
H200002495	COPS6-001
H300002242	TRIM7-001
opHsV0400013002	IL7R-001
opHsV0400000345	WDR1-013
H300021376	PPP1CA-001
opHsV0400000753	AC131180.1-201
H200002935	KIFC1-001
H200004731	INCENP-001
H200002802	CDK2-001
H300007069	SEMA7A-001
H200012822	EIF4A1-001
opHsV0400009148	EIF4A1-001
opHsV0400001722	TUBB-001
H200016720	KIF23-003
H200005936	TXNDC17-001
opHsV0400003249	SRSF3-001
H200019133	WDR18-201
H300003542	PRPS1-001
H200006992	TYMS-001
H200003422	CKAP2-001
H200002465	SNRNP25-001
H200006399	DNMT1-201
H300008046	TNFRSF11B-001
H200008448	SFPQ-001
H200006536	C1QBP-201
H200004166	CCDC99-001
opHsV0400001079	LINC00311-001
opHsV0400000984	COX8A-001
H300004324	ATP5G1-001
H200019650	TMEM141-001
H200005367	POC1A-001
H200002496	FANCD2-001
H200002187	TIPARP-001
H200002810	CRIM1-001
H200007728	SRSF3-001
H200011653	CORO1A-001
H200002746	NCAPG2-001
H300018868	ENPP1-001
H200007833	ALDH1B1-001
opHsV0400002502	TUBA1B-002
H200016523	CRISPLD2-001
opHsV0400001114	MYOZ1-001
H300015490	TRIM14-001
H200002701	ETS1-201
H300022614	TPM3-003

H200014262	RCAN1-001
H200005853	KIF20A-001
H200000897	CCDC86-001
H300019757	CSE1L-001
H200005718	FAM83D-001
opHsV0400001427	PPME1-001
H300011611	SNRPB-001
H300009386	ITGA5-001
H200015756	THOC4-201
opHsV0400008746	PGAM1-001
opHsV0400009836	RP11-295G20.2-001
opHsV0400000496	DDAH1-001
H200005643	SPHK1-201
H200008364	TUBB-001
H300015904	RANGAP1-001
opHsV0400005804	HMGN2-001
H300006274	TGM2-002
H200000680	LDHA-003
H200003216	NCEH1-201
opHsV0400006685	MT1JP-201
H200000919	FEN1-001
H200006364	CENPF-001
H200014823	GABBR2-001
opHsV0400006210	CAP1-001
H300010692	PHF19-001
H200005239	MCM2-001
H200010571	BAK1-002
opHsV0400006403	AP2S1-201
H300006714	VDAC1-002
H200001635	ESYT1-001
H200008648	ARL4D-201
H200005556	CLIC3-001
opHsV0400001657	CDCA4-001
H200003976	TRA2B-014
opHsV0400000164	HIST1H4B-001
H300017857	RFC2-001
H200007673	ATP8B1-001
opHsV0400003201	RHOC-001
H200005755	MRPS7-201
H200010373	PTPRN-001
H300016550	AURKAIP1-001
H300021724	RUVBL2-201
H200012190	H2AFZ-001
H200010050	AIMP2-001
H300016680	DEPDC1-001
H300019666	PRR11-201
H300005853	NHP2-001
H300017057	WNK4-201
H200012175	ACTN1-001
H200007498	TMEM2-001
H300000861	BOLA2-201

H200006246	GALE-001
H300013099	FAM64A-201
H200004829	GINS3-001
H200000191	FPGS-011
H200011039	ERCC2-001
H300017880	PLOD2-001
H200016159	TCF19-001
H200005811	CDA-001
H300003166	DCTPP1-001
opHsV0400001923	IDH2-001
opHsV0400002895	TPI1-001
H200017018	MRTO4-001
H200007046	ARPC2-001
H300019121	PRMT1-001
opHsV0400000259	TACO1-201
H200017078	TUBB6-001
H300001406	CNN1-201
H300013879	CCDC99-001
H300008232	WDR62-201
H300021167	SERINC2-001
opHsV0400000044	PSME2-001
H300020007	HNRNPA2B1-001
H300013153	E2F8-002
H200005726	HIST1H3H-001
H200019252	RUVBL1-001
H200005939	ITGA7-001
H200011336	HJURP-001
H200000490	GAL-001
H200005665	HAUS8-201
H300005482	NUDT1-001
H300010247	NUTF2-001
H300011776	SERINC2-001
H300003869	GPAT2-005
H200006317	LSM4-201
H200013298	RFC2-001
H200005976	PSMD2-001

Supplementary Table S5: Previous studies report widely different correlations between RNA-seq data and DNA microarray data.

Description of samples used	Reported correlation(s)	Reference(s)
Human embryonic kidney cells versus B cells	0.88	[7]
Human liver cells versus kidney cells	0.67 to 0.75	[4]
Yeast cells	0.099 to 0.509	[1, 5, 8]
Human brain cells	0.51 to 0.67	[2]
Male and female *Drosophila pseudoobscura*	0.54 to 0.94	[3]
Human T cells	0.79 to 0.90	[9]
Rat nerves	0.84 to 0.85	[6]
Human proliferating or quiescent fibroblasts	0.02 to 0.42	This study

Supplementary Table S6: Correspondence between the microarray data, the RNA-seq data, and the qRT-PCR data for the probes that were analyzed using qRT-PCR. These are the raw values used to construct Supplementary Figure S32.

Gene	$\log_2$ (QP1 FC)	$\log_2$ (QP2 FC)	$\log_2$ (QP3 FC)	$\log_2$ (QP4 FC)	Mean QP $\log_2$(FC)	$\log_2$(RNA-seq FC)	$\log_2$(qRT-PCR FC)
MAN1A1	3.37	2.23	3.56	2.50	2.91	1.63	3.00
MME	1.64	4.00	0.35	4.49	2.62	1.98	2.33
SSFA2	0.27	1.59	0.42	3.22	1.37	0.21	0.43
SOD2	1.09	2.31	0.95	2.73	1.77	2.16	2.97
TGFBR3	3.78	1.75	2.69	1.94	2.54	1.62	2.73
WISP2	-0.24	1.60	-0.71	1.01	0.41	4.71	4.44
PCSK9	1.32	0.37	1.33	0.00	0.75	3.48	4.64
SFRP2	0.70	0.07	0.30	-0.43	0.16	3.45	2.82
BMP4	0.79	-0.59	1.63	0.93	0.69	2.61	4.01
PODN	1.77	0.90	2.63	1.48	1.69	2.69	2.63
CTSK	2.26	1.82	2.02	1.69	1.95	4.66	5.17
SPRY1	-0.15	1.15	0.04	2.63	0.92	3.36	3.38
ECM2	1.76	1.10	1.98	1.33	1.54	2.19	0.69
GPRC5B	0.87	3.63	1.03	5.19	2.68	2.40	4.86
ADH1B	2.57	1.73	2.22	0.48	1.75	5.74	3.06
TNXB	2.68	2.37	1.27	1.14	1.87	5.58	3.01
APOD	6.00	9.68	2.54	5.79	6.00	5.33	5.61
PTGDS	0.94	3.84	0.52	3.83	2.28	4.79	3.62
FABP3	3.37	2.75	6.04	4.22	4.10	4.38	5.94
PPL	3.63	2.68	3.76	0.64	2.68	4.59	8.07
Col14A1	1.26	1.26	0.47	0.39	0.85	4.00	2.43
LTBP4	1.53	0.29	1.20	-1.15	0.47	4.08	3.07
EGR2	0.04	-0.64	0.07	-0.94	-0.37	3.44	1.01
C3	2.12	1.70	2.24	0.70	1.69	3.59	4.07
RARRES2	3.91	2.06	4.72	2.98	3.42	3.52	3.88
IL34	0.14	1.22	0.92	0.56	0.71	3.22	3.03
C2	1.02	0.88	2.44	1.21	1.39	3.29	1.55
IGF2	-0.28	-0.18	-0.22	-0.04	-0.18	2.96	0.55
C1R	3.31	3.01	2.37	3.15	2.96	2.80	2.06
IDI1	1.66	1.18	2.57	2.62	2.01	2.45	0.35
CFH	2.92	1.75	2.38	0.55	1.90	2.74	2.68
C1S	2.27	1.84	2.37	1.87	2.09	2.63	1.59
IFNA1	-0.66	0.64	-3.09	0.51	-0.65	2.29	1.21
APOE	2.10	1.47	1.85	0.27	1.42	4.28	3.98
GNG4	0.98	-0.54	-0.55	-0.41	-0.13	0.12	-0.64
RARb	0.10	-0.34	0.70	-0.34	0.03	0.01	1.59
TRPC1	0.74	-0.07	0.52	0.17	0.34	0.40	-5.35
TRPC6	0.25	1.42	-0.22	1.75	0.80	0.15	0.05
Tube1	0.51	-0.62	-0.31	0.51	0.02	-0.39	0.64
Gem	2.46	2.25	1.80	1.30	1.95	2.46	0.73

ActC1	-1.75	-1.36	-0.64	-2.50	-1.56	-3.08	0.77
IGIP	1.94	-0.19	1.41	0.72	0.97	1.69	3.02
SSTR3	0.03	-0.40	0.04	-0.53	-0.21	-0.01	-2.12
EDNRB	0.52	0.98	-0.57	0.22	0.29	0.32	-4.44
Nr4a1	0.47	0.46	-0.25	0.44	0.28	-0.07	-0.01
PTGS2	-3.61	0.49	-4.41	-0.58	-2.03	-2.17	3.48
B2M	-0.16	0.27	0.01	0.30	0.10	0.90	-1.84
LIF	-0.05	-0.05	0.17	-0.21	-0.03	-0.02	0.78
ActG2	-1.05	-2.20	-0.74	-2.39	-1.60	-2.15	2.08
Il8	-1.37	2.01	-2.14	0.79	-0.18	-0.28	1.94
EREG	0.11	1.67	-0.02	1.94	0.92	0.06	2.39
CXCL1	-1.21	1.86	-1.28	0.97	0.09	-0.69	1.14
Star	0.77	-0.10	0.01	-0.50	0.05	-0.68	0.23
SFN	0.66	-0.22	0.60	0.00	0.26	-1.06	-4.18
ITGa2	-0.62	0.03	-0.14	-0.66	-0.35	0.06	2.28
CLDN4	0.09	-0.10	0.50	-0.28	0.05	1.04	-3.47
THBD	-1.30	0.74	-0.20	0.57	-0.05	-1.31	-1.38
LAMB4	0.74	-0.27	0.14	-0.29	0.08	-0.03	-0.13
Spin2a	1.50	-0.12	0.17	-3.30	-0.44	0.66	0.52
NR4a2	0.42	4.99	-0.86	0.54	1.27	1.89	0.52
Jak2	-0.07	0.47	-0.03	0.34	0.18	0.25	-1.13
SATb1	0.48	-0.67	1.84	-0.78	0.22	0.71	-1.13
Sox4	-1.11	-0.46	0.06	-0.33	-0.46	2.01	-1.03
HIVEP1	-1.77	0.99	-0.91	0.84	-0.21	0.43	-0.14
Psg8	-0.97	-2.09	0.88	0.41	-0.44	-0.89	0.93
Lacc1	0.56	-0.24	0.67	-0.38	0.15	0.77	-1.84
Neat1	1.91	0.06	0.64	1.42	1.01	0.96	-1.35
Tp53	-0.25	-0.67	0.03	-0.57	-0.36	-0.47	2.35
Rit2	0.14	-0.20	-0.62	0.34	-0.08	0.01	2.34
ELMO1	0.46	-0.64	0.51	-0.83	-0.13	0.02	-0.83
TGFA	0.22	-0.48	0.00	-0.87	-0.28	-0.28	0.96
Malat1	1.29	-0.66	0.35	0.26	0.31	0.18	-0.05
FAM180a	-0.57	2.46	-0.78	2.47	0.90	1.10	0.60
FADS3	-0.52	0.31	-0.71	0.08	-0.21	-1.33	-0.05
PRSS1	0.35	-0.41	0.69	-1.64	-0.25	0.00	4.86
PREPL	0.44	0.82	0.08	0.82	0.54	0.35	0.75

Supplementary Table S7: Parameter values used for SeqMonk.

Parameter name	Value
**Importing data**	
Remove duplicate reads	no
Treat as HiC data	no
Min mapping quality	0
Split spliced reads	yes
Import introns rather than exons	no
Data type	Paired end
Pair distance cutoff (bp)	1000
**Generating probes**	
Type Feature	probe generator
Features to design around	mRNA
Split into subfeatures (exons)	no
Remove exact duplicates	no
Ignore feature strand information	no
Make probes	Over feature
**Read count quantitation**	
Count reads on strand	All reads
Corect for total read count	yes
Correct to what?	Per million reads
Count total only within probes	no
Correct for probe length	yes
Log transform count	no
Count duplicated reads only once	no
Only quantitate visible stores	no

References

[1] L David, W Huber, M Granovskaia, J Toedling, C J Palm, L Bofkin, T Jones, R W Davis, and L M Steinmetz. A high-resolution map of transcription in the yeast genome. *Proc Natl Acad Sci U S A*, 103(14):5320–5, 2006.

[2] X Fu, N Fu, S Guo, Z Yan, Y Xu, H Hu, C Menzel, W Chen, Y Li, R Zeng, and P Khaitovich. Estimating accuracy of RNA-Seq and microarrays with proteomics. *BMC Genomics*, 10:161, 2009.

[3] J H Malone and B Oliver. Microarrays, deep sequencing and the true measure of the transcriptome. *BMC Biol*, 9:34, 2011.

[4] J C Marioni, C E Mason, S M Mane, M Stephens, and Y Gilad. RNA-seq: an assessment of technical reproducibility and comparison with gene expression arrays. *Genome Res*, 18(9):1509–17, 2008.

[5] U Nagalakshmi, Z Wang, K Waern, C Shou, D Raha, M Gerstein, and M Snyder. The transcriptional landscape of the yeast genome defined by RNA sequencing. *Science*, 320(5881):1344–9, 2008.

[6] J R Perkins, A Antunes-Martins, M Calvo, J Grist, W Rust, R Schmid, T Hildebrandt, M Kohl, C Orengo, S B McMahon, and D L H Bennett. A comparison of RNA-seq and exon arrays for whole genome transcription profiling of the L5 spinal nerve transection model of neuropathic pain in the rat. *Mol Pain*, 10:7, 2014.

[7] Marc Sultan, Marcel H Schulz, Hugues Richard, Alon Magen, Andreas Klingenhoff, Matthias Scherf, Martin Seifert, Tatjana Borodina, Aleksey Soldatov, Dmitri Parkhomchuk, Dominic Schmidt, Sean O'Keeffe, Stefan Haas, Martin Vingron, Hans Lehrach, and Marie-Laure Yaspo. A global view of gene activity and alternative splicing by deep sequencing of the human transcriptome. *Science*, 321(5891):956–60, 2008.

[8] Z Wang, M Gerstein, and M Snyder. RNA-Seq: a revolutionary tool for transcriptomics. *Nat Rev Genet*, 10(1):57–63, 2009.

[9] S Zhao, W-P Fung-Leung, A Bittner, K Ngo, and X Liu. Comparison of RNA-Seq and microarray in transcriptome profiling of activated T cells. *PLoS One*, 9(1):e78644, 2014.
